# Low-Temperature Dyeing of Chemically Modified PET/Spandex Blends: A Sustainable Approach for Enhanced Dyeability and Color Fastness †

**DOI:** 10.3390/molecules30173578

**Published:** 2025-09-01

**Authors:** Md Morshedur Rahman, Nazrul Hsan, Ingi Hong, Shekh Md Mamun Kabir, Seunga Choi, Youngdae Kim, Soohyun Kim, Joonseok Koh

**Affiliations:** 1 Advanced Materials Program, Department of Materials Science and Engineering, Konkuk University, Seoul 05029, Republic of Korea; morshed133@gmail.com (M.M.R.); modi7660@konkuk.ac.kr (S.K.); 2 Department of Materials Science and Engineering, Konkuk University, Seoul 05029, Republic of Korea; 3 Department of Textile Materials Engineering, Bangladesh University of Textiles, Dhaka 1208, Bangladesh

**Keywords:** polyester/spandex blends, poly(ethylene terephthalate-co-polyethylene glycol), disperse dyeing, low-temperature dyeing, color fastness

## Abstract

Blends of polyethylene terephthalate (PET) with spandex are widely used in sportswear and outdoor apparel. However, dyeing PET/spandex fabrics remains challenging due to the high energy required at elevated dyeing temperatures and persistent problems with poor color fastness caused by dye staining on the spandex component. In this study, we investigated the dyeing behavior of a chemically modified poly(ethylene terephthalate-co-polyethylene glycol) (PCP) blended with spandex and compared it with conventional PET/spandex blends. The PCP/spandex fabrics exhibited significantly improved dyeability, showing higher dyebath exhaustion and greater color strength than PET/spandex blends, particularly at sub-conventional dyeing temperatures. The optimal dyeing condition for PCP/spandex blends was identified as 110 °C for 60 min, which provided a balance between enhanced dye uptake and minimized spandex staining. Moreover, PCP/spandex fabrics demonstrated improved color fastness at lower dyeing temperatures (110–120 °C), primarily due to the reduced staining tendency of the spandex component when blended with PCP fibers. This reduction in spandex staining minimized dye migration during washing. Overall, these findings suggest that PCP/spandex blends offer a promising, energy-efficient alternative to conventional PET/spandex fabrics. They enable effective dyeing at lower temperatures while achieving improved color fastness, thereby addressing key challenges in the dyeing of elastic fiber blends.

## 1. Introduction

Elastomeric fibers such as spandex have attracted increasing attention in recent years, owing to their unique stretch and recovery properties [1,2,3,4,5,6]. Spandex is primarily composed of polyurethane, which features a segmented structure with alternating soft and hard domains. This architecture imparts excellent elasticity and resilience to the fiber [7,8,9]. To enhance comfort and performance, spandex is often blended with other fibers in the production of garments and technical textiles requiring high stretchability. Common applications include undergarments, socks, bodices, corsets, sportswear, swimwear, hosiery, tights, ribbons, medical stockings, bandages, and disposable hygiene products [10]. Although the proportion of spandex in these blends is generally low, its presence significantly improves the comfort, fit, and functionality of the final product [11]. Among the various fiber combinations, polyethylene terephthalate (PET)/spandex blends are especially prevalent, particularly in sportswear and outdoor apparel—sectors that continue to grow with the global rise in active lifestyles and sports participation [12,13,14,15]. Consequently, the dyeing of PET/spandex blended fabrics has become increasingly important in the global textile market. Owing to the hydrophobic nature of both PET and spandex, disperse dyes—non-ionic colorants particularly suited for synthetic fibers—are typically employed [4].

However, dyeing PET/spandex blends with disperse dyes presents two major technical challenges: (1) the inherently low dyeability of PET and (2) severe dye staining on the spandex component [16,17]. To achieve adequate dye uptake, PET fibers are typically dyed at elevated temperatures (≈130 °C) [18,19,20,21]. Such conditions, however, can cause thermal degradation of spandex, leading to a loss of its elastic recovery properties. This limitation necessitates lowering the dyeing temperature (generally below 125 °C), which unfortunately reduces dye diffusion into the PET matrix and results in insufficient shade depth [22,23]. In contrast, spandex fibers—characterized by a glass transition temperature (Tg) below room temperature—readily absorb disperse dyes, causing excessive staining [14]. During subsequent heat-setting, these absorbed dyes migrate toward the fiber surface, and during laundering, they are easily released, producing a pronounced decline in wash fastness.

**Figure 1:**
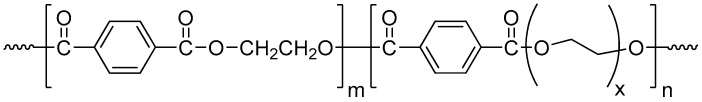
Chemical structure of poly(ethylene terephthalate-co-polyethylene glycol) fiber [1].

To address the challenges associated with dyeing PET/spandex blends, several strategies have been proposed in the literature. These include the selection of suitable commercial disperse dyes [14,16,17,22,23], the design and synthesis of novel disperse dyes [11,15,24,25], and the application of clearing processes to remove surface-bound dyes from the spandex component [4,14]. Among these, one of the most promising approaches is the chemical modification of PET fibers through copolymerization with soft segment comonomers. This modification introduces a more open molecular structure, thereby enhancing dye diffusion and enabling improved dye uptake at lower temperatures [26,27,28]. A representative example of such modified polyesters is poly(ethylene terephthalate-co-polyethylene glycol) (PCP), produced by incorporating polyethylene glycol (PEG) segments into the PET backbone (Figure 1) [1,27]. This structural adjustment classifies PCP as an easy-dyeable polyester [28]. Despite the inclusion of PEG units, the fundamental polymeric characteristics of PET are preserved. PCP has demonstrated the ability to achieve effective dyeing at significantly lower temperatures than conventional PET, with optimal dyeability reported around 100 °C [27,29]. This enhanced dyeability under milder thermal conditions highlights its potential for substantial energy savings through atmospheric-pressure dyeing. Furthermore, the higher dye uptake of PCP is expected to reduce spandex staining in PCP/spandex blends, thereby improving the overall color fastness of the final fabric.

In this study, the dyeing behavior of PCP/spandex blend fabrics was systematically investigated using two disperse dyes with different molecular weights: C.I. Disperse Red 60 (Red 60, MW = 331.32), classified as a low-energy dye, and C.I. Disperse Red 167 (Red 167, MW = 505.91), classified as a high-energy dye. Key dyeing parameters—including color strength (K/S value), dyebath exhaustion, distribution ratio, and kinetic characteristics—were evaluated across a temperature range of 90–130 °C. For comparison, the same set of analyses was performed on conventional PET/spandex blend fabrics, enabling a direct assessment of the relative performance of PCP/spandex versus PET/spandex systems.

## 2. Results and Discussion

### 2.1. Dyeing Behaviors of Polyesters/Spandex Fabrics in Terms of Color Strength

**Figure 2:**
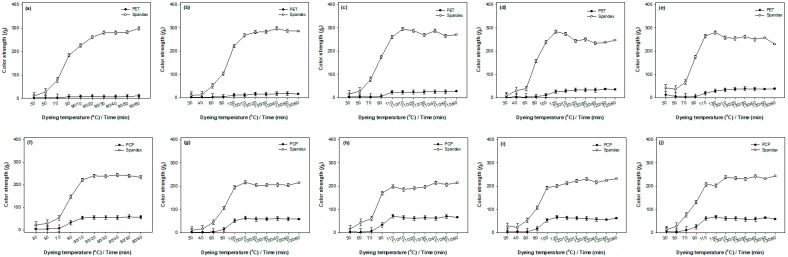
Color strength of polyesters and staining on spandex in polyester/spandex blends dyed with Red 60 at different target temperatures in the same dyebath: (**a**–**e**) PET/spandex and (**f**–**j**) PCP/spandex. Values are mean ± SD (*n* = 3; error bars = SD).

**Figure 3:**
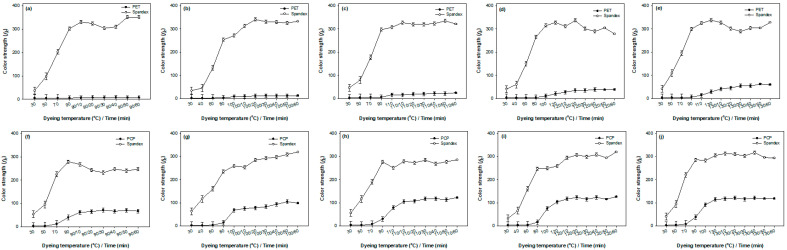
Color strength of polyesters and staining on spandex in polyester/spandex blends dyed with Red 167 at different target temperatures in the same dyebath: (**a**–**e**) PET/spandex and (**f**–**j**) PCP/spandex. Values are mean ± SD (*n* = 3; error bars = SD).

Figure 2 and Figure 3 present the color strength of PET and PCP components, as well as the staining behavior of the spandex component, in PET/spandex and PCP/spandex blends dyed with Red 60 and Red 167 across different dyeing temperatures (90, 100, 110, 120, and 130 °C). The corresponding visual images of the dyed fabrics are provided in Tables S1–S4.

The results presented in Figure 2 and Figure 3 indicate that the color strength of the polyester components and the staining of the spandex component followed a characteristic pattern: an initial lag phase, a subsequent rapid increase, and a final plateau. This behavior is well described by a sigmoidal (S-shaped) model [30]. In this study, the experimental data (Figure 2 and Figure 3) were fitted using a four-parameter logistic (sigmoidal) regression model (Figures S1 and S2), as expressed in Equation (1) [31]. The estimated logistic parameters are summarized in Tables S5–S8. The model provided an excellent fit to the color strength data, as demonstrated by R^2^ and adjusted R^2^ values approaching 1. Moreover, *p* values greater than 1 indicated that the curve steepens considerably around the midpoint ($x_{0}$).

(1) $$y=A_{2}+\frac{\left(A_{1}-A_{2}\right)}{1+{\left(x/x_{0}\right)}^{p}}$$

where y = dependent variable (e.g., color strength), *x* = independent variable (e.g., dyeing time at different temperatures), $A_{1}$ = value of *y* at the start, $A_{2}$ = value of y at the end (maximum value), $x_{0}\;$= the value of x at which y reaches half of the maximum value, and p = slope (steepness of the curve).

The experimental data reveal distinct differences in dyeing behavior between PET/spandex and PCP/spandex blends. For PET blended with spandex, color strength remained notably low at 90 and 100 °C, even after 60 min of dyeing (Figure 2 and Figure 3) (90 and 100 min total, Figure S1a,b). This limited dye uptake can be attributed to the restricted segmental mobility of PET at these temperatures, which hinders dye diffusion into the polymer matrix. As the dyeing temperature increased to 110, 120, and 130 °C, a progressive enhancement in color strength was observed, reflecting the increased chain mobility of PET that facilitates greater dye penetration [21]. The most pronounced increase occurred at 130 °C, where maximum color strength was achieved at 20 min for Red 60 (70 min total, Figure S1e) and 50 min for Red 167 (100 min total, Figure S2e). The longer dyeing time required for Red 167 is attributed to its larger molecular size, which slows diffusion and necessitates extended dyeing times for sufficient uptake. In contrast, PCP/spandex blends demonstrated appreciable color strength even at 90 °C, with effective dye uptake observed within just 10 min (Figure 2 and Figure 3) (40 min total, Figures S1f and S2f). This superior dyeability at lower temperatures is attributed to the incorporation of flexible comonomers in the PCP structure (Figure 1), which increase segmental mobility and thereby promote efficient dye diffusion under reduced thermal conditions [27,29]. A marked increase in color strength was observed between 90 and 120 °C. For Red 60, maximum color strength was reached at 110 °C immediately after attaining the target temperature (40 min total, Figure S1h), whereas for Red 167, the maximum occurred at 110 °C after 30 min (70 min total, Figure S2h). At 130 °C, however, a slight decline in color strength was recorded, suggesting the onset of dye desorption from the PCP matrix [29]. These findings confirm that PCP fibers exhibit substantially better dyeability than conventional PET fibers at sub-conventional dyeing temperatures (<130 °C) when using disperse dyes. This conclusion is further supported by statistical analysis. As shown in Tables S5 and S6, the logistic parameter A_2_, which represents maximum color strength, increased gradually for PET, reaching its peak at 130 °C. By contrast, Tables S7 and S8 indicate that PCP achieved maximum A_2_ values at 110 °C for Red 60 and 120 °C for Red 167, followed by a decline at 130 °C for both dyes.

The spandex component in PET or PCP blends exhibited noticeable staining (Tables S1–S4). This behavior is primarily attributed to the intrinsically low Tg of spandex, which lies below room temperature. The resulting high free volume facilitates rapid dye absorption [32]. The extent of staining was strongly dependent on dyeing temperature. In PET/spandex blends dyed at 90 and 100 °C, the limited dye uptake by PET left excess dye in the bath, which was readily absorbed by the more permeable spandex, resulting in pronounced staining (Figure 2 and Figure 3; Tables S1 and S2). As the dyeing temperature increased to 110–130 °C, PET dye uptake improved, reducing dye availability for spandex and consequently lowering staining. At 130 °C, PET achieved maximum color strength with minimal spandex staining; however, this condition is not optimal due to the risk of spandex degradation at elevated temperatures. In contrast, PCP/spandex blends displayed distinct dyeing behavior, largely due to the lower Tg of PCP [27]. The enhanced chain mobility of PCP at lower temperatures (e.g., 90 °C) facilitated efficient dye uptake, as reflected by higher color strength values (Figure 2 and Figure 3). This reduced residual dye in the bath and limited staining of spandex. With increasing temperatures (100–120 °C), dye diffusion into the PCP matrix was further promoted, enabling dye migration from spandex back into PCP. At 130 °C, however, a slight decline in PCP color strength indicated dye desorption, which reintroduced dye into the bath and caused a modest increase in spandex staining. These findings suggest that optimal dyeing conditions for PCP/spandex blends are achieved below 130 °C, where efficient dye uptake by PCP is coupled with minimal spandex staining and reduced risk of thermal degradation. These experimental observations are consistent with the statistical analysis. For PET/spandex blends (Tables S5 and S6), spandex showed higher A_2_ values at 90–100 °C, reflecting heavier staining, which decreased progressively with increasing temperature. In PCP/spandex blends (Tables S7 and S8), lower A_2_ values were observed at 90–110 °C, indicating reduced spandex staining; but values increased again at 130 °C due to dye desorption. Moreover, across all blends, spandex stained more rapidly than the polyester components, as evidenced by lower x_0_ values in the logistic regression analysis.

It was further observed that Red 167 consistently produced deeper shades, with higher color strength values, on both PET and PCP compared to Red 60 (Figure 2 and Figure 3; Tables S1–S4). This difference can be attributed to the intrinsic tinctorial strength of the azo chromophore in Red 167, which is generally greater than that of the anthraquinone chromophore in Red 60 [33]. As a result, the color strength values of Red 167 increased more markedly with extended dyeing times at all temperatures, relative to Red 60. This trend was corroborated by the statistical analysis, which showed consistently higher A_2_ values for Red 167 compared with Red 60 (Tables S5–S8).

In summary, this study demonstrates a clear inverse relationship between color strength on PET or PCP and the degree of staining on spandex. PCP consistently exhibited superior dyeability compared to PET, resulting in reduced spandex staining, particularly at dyeing temperatures below 130 °C. By balancing the objectives of maximizing color strength, minimizing spandex staining, and preserving fiber integrity, a dyeing temperature of 110 °C is proposed as optimal for PCP/spandex blends with both disperse dyes examined. It should be noted, however, that color strength is typically evaluated through reflectance measurements of selected fabric surface areas, which may not fully represent the actual extent of dye absorption during the dyeing process [29,34]. To provide a more comprehensive evaluation of dye uptake behavior in PET/spandex and PCP/spandex blends, the next section examines dyebath exhaustion as a function of dyeing temperature.

### 2.2. Dyeing Behaviors of Polyesters/Spandex Fabrics in Terms of Exhaustion (%)

The dyebath exhaustion of the polyester component (80%)—either PET or PCP—and the staining of the spandex component (20%) in PET/spandex (80:20) and PCP/spandex (80:20) blends were evaluated as a function of dyeing temperature (90, 100, 110, 120, and 130 °C). The corresponding results are presented in Figures S3 and S4. These experimental data (Figures S3 and S4) were further fitted to Equation (1), where y (the dependent variable) represents the exhaustion percentage. The estimated logistic fit parameters derived from the model are summarized in Tables S9–S12.

For Red 60, PET/spandex blends exhibited a pronounced contrast between dye exhaustion on PET and staining on spandex at lower dyeing temperatures (90 and 100 °C). At these conditions, heavy staining was observed on the spandex component (Figure S3), attributable to the limited segmental mobility of PET, which restricted dye uptake and left more dye available for spandex absorption. Specifically, at 90 and 100 °C, PET showed only 20–30% dyebath exhaustion, while spandex staining was considerable. As the dyeing temperature increased to 110 and 120 °C, PET exhaustion rose to ~50%, accompanied by reduced spandex staining. At 130 °C, PET exhibited significantly enhanced segmental mobility, achieving ~60% exhaustion, with a marked decrease in spandex staining. This shift reflects a redistribution of dye affinity from spandex to PET as the temperature increased [14,17]. The crossover point—where PET exhaustion exceeded spandex staining—occurred 20 min after reaching 120 °C (70 min total, Figure S3d) and immediately after reaching 130 °C (50 min total, Figure S3e). In contrast, PCP/spandex blends consistently showed higher dye exhaustion on PCP across all dyeing temperatures (Figure S3). At 90 °C, PCP achieved ~70% exhaustion, with relatively low spandex staining. Between 100 and 120 °C, PCP exhaustion stabilized at ~80%, while spandex staining further decreased. At 130 °C, however, PCP exhaustion declined slightly (<80%), with a modest increase in spandex staining, attributable to dye desorption from the PCP matrix, as previously discussed. Notably, spandex staining in PCP/spandex blends remained consistently lower than in PET/spandex blends throughout the entire temperature range. These trends were corroborated by statistical analysis. At lower temperatures (90–100 °C), PET exhibited lower exhaustion (lower A_2_ values), while spandex showed heavier staining (higher A_2_ values; Table S9). With increasing temperature, PET exhaustion improved (higher A_2_ values), while spandex staining decreased (lower A_2_ values). By contrast, PCP showed higher exhaustion up to 120 °C (higher A_2_ values; Table S11), followed by a decline at 130 °C (lower A_2_ values). Spandex blended with PCP exhibited lower staining at 100 and 110 °C (lower A_2_ values), but relatively higher staining at 90, 120, and 130 °C (higher A_2_ values). Overall, however, spandex in PCP blends consistently stained less than spandex in PET blends, as reflected by consistently lower A_2_ values (Tables S9 and S11).

For Red 167, PET/spandex blends exhibited substantially higher spandex staining at 90 and 100 °C, while PET exhaustion remained very low (~10%) (Figure S4). Even at 110 °C, PET exhaustion increased only modestly (~20%), with spandex staining still prominent. A marked shift occurred at 120 °C, where spandex staining decreased, and PET exhaustion rose to ~40%. At 130 °C, PET exhaustion reached ~50%, accompanied by the lowest level of spandex staining. The crossover point—where PET exhaustion exceeded spandex staining—was observed at 130 °C after 40 min of dyeing (90 min total, Figure S4e). In contrast, PCP/spandex blends dyed with Red 167 displayed distinctly different exhaustion behavior (Figure S4). At 90 °C, PCP exhaustion was already ~60%, though accompanied by slightly higher spandex staining. As the dyeing temperature increased to 100 °C, PCP exhaustion rose to ~70%, with minimal staining, a trend that remained stable through 120 °C. At 130 °C, PCP exhaustion decreased slightly to below 80%, while spandex staining increased modestly. Importantly, across all dyeing conditions, PCP/spandex blends consistently exhibited lower spandex staining than PET/spandex blends. These experimental observations align with the predicted exhaustion values (A_2_) from the logistic model (Tables S10 and S12). However, it should be noted that PET exhaustion behavior at 90 and 100 °C showed poor agreement with the model, attributable to the minimal dye uptake by PET at these temperatures (Figures S3 and S4).

Among the dyes investigated, Red 60 exhibited higher exhaustion on both polyester components (PET and PCP) compared with Red 167 (Figures S3 and S4). This behavior can be attributed to the smaller molecular size of Red 60, which facilitates more efficient diffusion and uptake into the polyester matrix [29,35]. By contrast, the larger molecular size of Red 167 restricted its diffusion into polyester, resulting in lower exhaustion values. Consequently, Red 167 remained in the dyebath for a longer period, increasing its availability for absorption by spandex, which possesses a higher free volume. This trend is consistent with the statistical analysis, as reflected by the higher A_2_ values for spandex with Red 167 relative to Red 60 (Tables S9–S12).

In summary, the results strongly support the hypothesis that greater dye uptake by the polyester component correlates with reduced staining on spandex. PCP consistently demonstrated superior dyeability, achieving dyebath exhaustion levels of approximately 80%. This enhanced performance is attributed to the incorporation of flexible comonomers in its structure, which increase segmental mobility and promote dye diffusion. The improved dye uptake of PCP plays a critical role in minimizing dye staining on spandex. Taken together, these findings indicate that dyeing at 110 °C for 60 min represents the optimal condition for PCP/spandex blends with both dyes, consistent with the color strength results. To further elucidate dye partitioning between polyester (PET or PCP) and spandex within the blends, distribution ratios were evaluated and are discussed in the following section.

### 2.3. Kinetics Analysis of Disperse Dyes on Polyesters (PET or PCP) and Spandex Fabrics

In general, two primary approaches are employed to investigate dyeing mechanisms: thermodynamic and kinetic analyses. Because commercial dyeing processes are rarely carried out to equilibrium, greater emphasis is typically placed on kinetic analysis, particularly, the rate at which dye molecules diffuse into textile fibers. This rate is most commonly characterized by the diffusion coefficient (D) of the dye within the fiber. The diffusion coefficient is a critical parameter that reflects the ease of dye migration through the fiber matrix; a higher D value indicates lower resistance to diffusion and thus faster dye penetration. Several factors influence the diffusion coefficient, including the initial dye concentration, dyeing time, dyeing temperature, fiber type, and yarn count [29].

**Figure 4:**
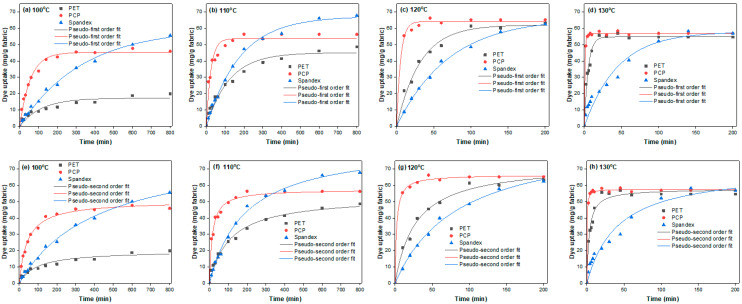
Dyeing kinetics curves for Red 60 on PET, PCP, and spandex fibers at different temperatures: (**a**–**d**) pseudo-first order and (**e**–**h**) pseudo-second order fitting.

**Figure 5:**
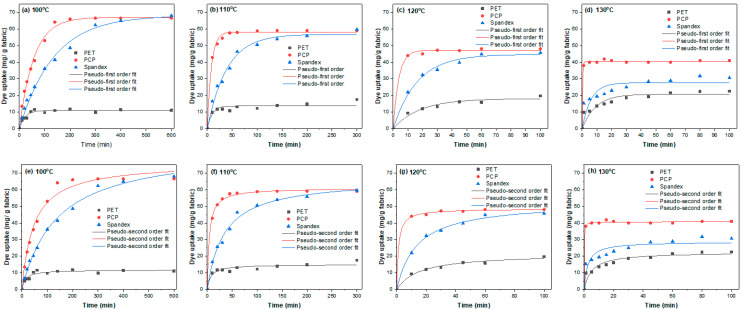
Dyeing kinetics curves for Red 167 on PET, PCP, and spandex fibers at different temperatures: (**a**–**d**) pseudo-first order and (**e**–**h**) pseudo-second order fitting.

In this study, the diffusion coefficients of Red 60 and Red 167 on PET, PCP, and spandex fibers were evaluated. Dyeing rate curves were obtained for each fiber at 100, 110, 120, and 130 °C until equilibrium was reached (Figure 4 and Figure 5). Equilibrium was defined as the point at which prolonged dyeing produced no further increase in dye uptake. Dyeing at 90 °C was excluded from the kinetic analysis because the equilibrium time for PET fibers at this temperature was excessively long and could not be reliably achieved using the dyeing apparatus. The experimental dyeing rate data were subsequently fitted to pseudo-first-order and pseudo-second-order kinetic models (Figure 4 and Figure 5). The model that provided the best fit was selected to determine the half dyeing time (t_1_/_2_), which was then used to calculate the D according to Equation (8).

Figure 4 and Figure 5 show that the dyeing rate increased with rising temperature. However, equilibrium dye uptake decreased at higher temperatures, consistent with the exothermic nature of textile dyeing. Both pseudo-first-order and pseudo-second-order kinetic models provided good agreement with the experimental data; however, based on the coefficient of determination (R^2^ > 0.992), the dyeing process was best described by the pseudo-second-order model. The t_1/2_ obtained from this model was subsequently used to calculate the D. As summarized in Table 1, spandex fibers exhibited relatively higher D values than both PET and PCP, reflecting the more open amorphous structure of spandex compared with polyesters. Interestingly, although spandex displayed higher D values, the kinetic curves (Figure 4 and Figure 5) revealed a steeper initial slope for PCP, suggesting a faster initial dyeing rate. From a kinetic standpoint, PCP would therefore be expected to exhibit higher diffusion coefficients than spandex. The observed variation can be explained by yarn characteristics: spandex has a lower density (0.96 g/cm^3^) than PCP (1.37 g/cm^3^), and since D is quadratically related to fiber diameter (Equation (8)), the lower density and larger effective radius of spandex fibers contribute to their higher D values. When comparing PCP with PET, which have comparable densities, the higher D values for PCP are attributed to the presence of flexible copolymer segments that enhance segmental mobility and facilitate dye diffusion. Finally, between the two dyes studied, Red 60 exhibited lower D values than Red 167, which can be explained by its longer t_1/2_.

Overall, the kinetic analysis indicates that the higher diffusion coefficients of spandex facilitate rapid dye penetration into the fiber but also increase the likelihood of dye bleeding, which can compromise the fastness properties of the final products. The results further demonstrate that PCP exhibits higher diffusion coefficients than PET, and in PCP/spandex blends, these higher D values enable PCP to absorb a greater proportion of dye from the dyebath, thereby limiting dye uptake by spandex. This finding is consistent with the trends observed in the color strength and exhaustion analyses.

### 2.4. Comparative Analysis of Disperse Dye Distribution in Polyester/Spandex Blend Fabrics

**Figure 6:**
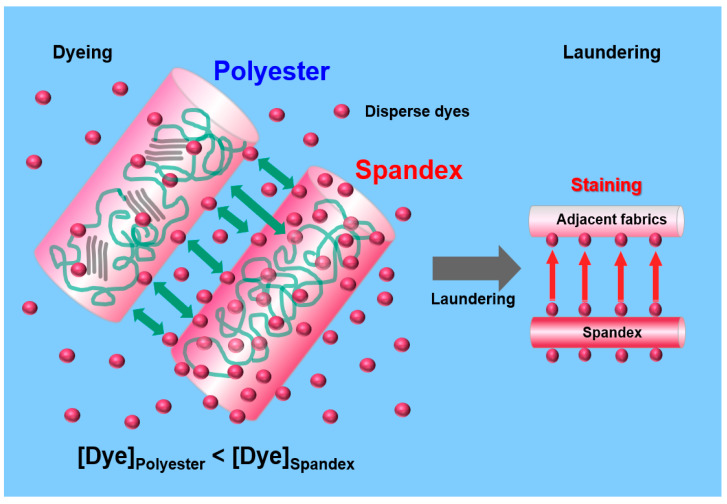
Distribution behavior of disperse dyes in polyester/spandex blends and its impact on color fastness.

In the dyeing of polyester/spandex blend fabrics, the extent to which disperse dyes partition between the spandex and polyester components plays a decisive role in determining the color fastness of the final product. Polyester, with its relatively high degree of crystallinity, absorbs dyes primarily through the generation of free volume at elevated temperatures above its Tg (70–80 °C) [8,21,33]. By contrast, spandex, composed predominantly of amorphous regions, can absorb dyes more readily even at lower temperatures due to its high chain mobility and associated free volume [32] (Figure 6). However, the same amorphous structure that facilitates dye uptake also increases the likelihood of dye desorption. Consequently, greater dye staining on the spandex component raises the risk of poor wash fastness in polyester/spandex fabrics, as dyes are prone to bleeding during laundering or wear. From this perspective, evaluating the distribution ratio of dye between polyester and spandex is of critical importance for understanding and improving the dyeing performance of such blends.

**Figure 7:**
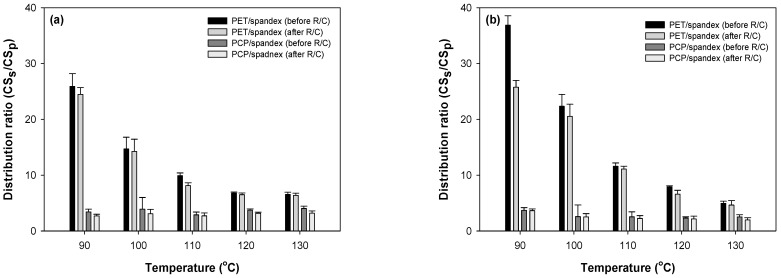
Distribution ratio of disperse dyes between spandex and polyester (PET or PCP) fabrics evaluated by color strength at different dyeing temperatures: (**a**) Red 60 and (**b**) Red 167.

**Figure 8:**
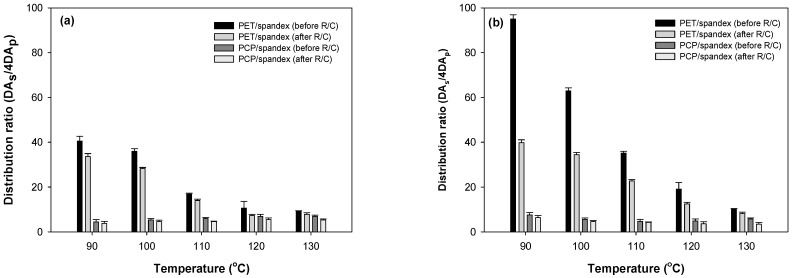
Distribution ratio of disperse dyes between spandex and polyester (PET or PCP) fabrics evaluated by amount of dyes extracted at different dyeing temperatures: (**a**) Red 60 and (**b**) Red 167.

The distribution of disperse dyes—expressed as the ratio of dye uptake in spandex to that in polyester (dye uptake in spandex/dye uptake in polyester)—was evaluated for PET/spandex and PCP/spandex blends. Two types of distribution ratios were calculated: one based on the color strength values of the dyed fabrics and the other based on the quantity of dye extracted from the samples (Equation (9)). Both distribution ratios were determined before and after the R/C process for Red 60 and Red 167, as illustrated in Figure 7 and Figure 8, respectively.

The results show that PET/spandex blends exhibited higher distribution ratio values, indicating more pronounced staining of the spandex component when blended with PET (Figure 7 and Figure 8). At lower dyeing temperatures (90 and 100 °C), heavy staining on spandex was observed, consistent with earlier findings. As the dyeing temperature increased to 110–130 °C, the distribution ratio decreased, reflecting reduced spandex staining and enhanced dye uptake by PET, with the lowest ratio recorded at 130 °C. The R/C process further decreased the distribution ratio in PET/spandex blends, confirming that dye removal was more effective from spandex than from PET. In contrast, PCP/spandex blends consistently exhibited lower distribution ratio values across all dyeing temperatures, demonstrating that spandex staining was substantially suppressed (Figure 7 and Figure 8). This behavior is attributed to the incorporation of flexible copolymer segments in PCP, which promote greater dye uptake by the polyester component and thereby limit dye adsorption on spandex. A slight increase in the distribution ratio was observed at 130 °C, likely due to dye desorption from PCP, resulting in minor staining of spandex. Similar to PET/spandex blends, the R/C process reduced the distribution ratio in PCP/spandex blends, further confirming diminished spandex staining after treatment.

Among the two dyes examined, Red 167 consistently produced higher levels of staining on the spandex component (Figure 7 and Figure 8). Both methods of calculating the distribution ratio revealed consistent trends: spandex in PET/spandex blends exhibited greater staining than spandex in PCP/spandex blends. These findings are in agreement with earlier observations from the color strength and dyebath exhaustion analyses.

In conclusion, the incorporation of PCP into spandex blends significantly reduces dye staining on the spandex component, particularly at a dyeing temperature of 110 °C, which was identified as the optimal condition. This suppression of spandex staining is expected to enhance the overall color fastness of PCP/spandex fabrics compared with conventional PET/spandex blends, in which spandex tends to stain heavily under similar dyeing conditions.

### 2.5. Build-Up Properties of Polyesters (PET or PCP) and Spandex Fabrics

**Figure 9:**
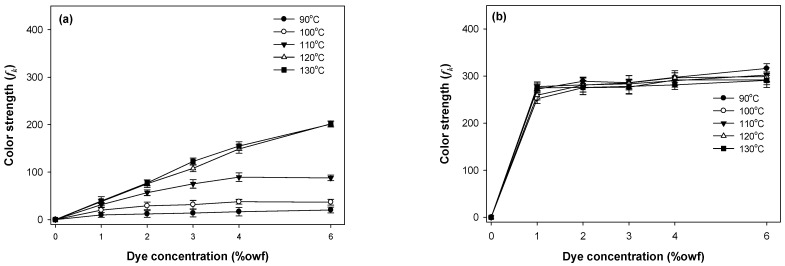
Color strength of (**a**) PET and (**b**) spandex in PET/spandex (80:20) blends dyed in the same dyebath with Red 60 at varying temperatures and dye concentrations.

**Figure 10:**
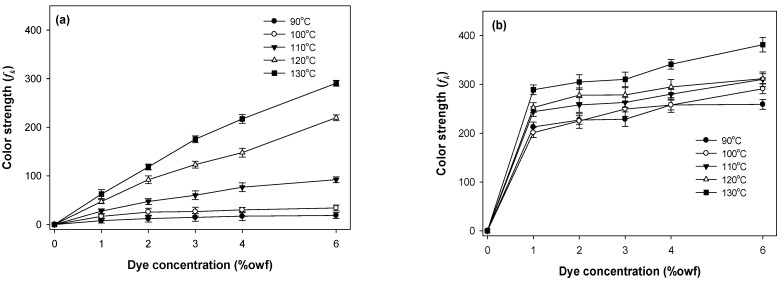
Color strength of (**a**) PET and (**b**) spandex in PET/spandex (80:20) blends dyed in the same dyebath with Red 167 at varying temperatures and dye concentrations.

**Figure 11:**
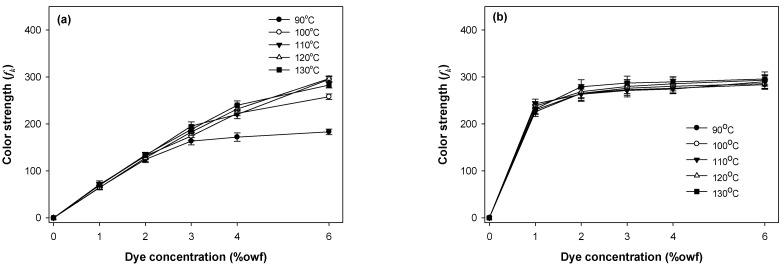
Color strength of (**a**) PCP and (**b**) spandex in PCP/spandex (80:20) blends dyed in the same dyebath with Red 60 at varying temperatures and dye concentrations.

**Figure 12:**
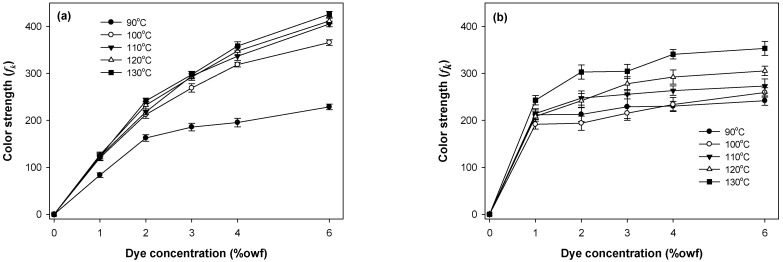
Color strength of (**a**) PCP and (**b**) spandex in PCP/spandex (80:20) blends dyed in the same dyebath with Red 167 at varying temperatures and dye concentrations.

The build-up properties of the polyester components (PET or PCP) and spandex in PET/spandex and PCP/spandex blends were investigated using the disperse dyes Red 60 and Red 167 under varying dyeing temperatures and dye concentrations, as illustrated in Figure 9, Figure 10, Figure 11 and Figure 12.

For both dyes, PET exhibited a consistent increase in color strength with rising dye concentration. Figure 9 and Figure 10 [25,36,37,38,39]. Dyeing temperature exerted a significant influence on color development: as the temperature increased from 90 °C to 130 °C, color strength rose markedly across all concentrations, reaching maximum values at 130 °C. This enhancement is attributed to the increased free volume generated within the PET matrix at elevated temperatures, which facilitates greater dye diffusion. PCP displayed a similar trend with increasing dye concentration; however, its color strength was significantly higher than that of PET under all conditions (Figure 11 and Figure 12). The effect of dyeing temperature on PCP was less pronounced. While color strength increased noticeably between 90 °C and 100 °C, further increases up to 130 °C resulted in only marginal gains. This behavior reflects the inherently superior dyeability of PCP, particularly at sub-conventional temperatures (<130 °C). Notably, dyeing at 110 °C produced color strength values comparable to those obtained at higher temperatures, consistent with previously reported optimal conditions for PCP. Between the two dyes, Red 167 consistently yielded higher color strength than Red 60 across all dye concentrations and dyeing temperatures.

With respect to the staining behavior of the spandex component, saturation was observed at dye concentrations of approximately 1% owf (Figure 9, Figure 10, Figure 11 and Figure 12). Beyond this concentration, further increases produced only marginal gains in staining. Dyeing temperature exerted minimal influence on spandex staining, reflecting the inherently high free volume of spandex, which facilitates dye absorption even at room temperature. Notably, spandex blended with PCP exhibited slightly lower staining than spandex blended with PET. Among the dyes studied, Red 167 produced more pronounced staining on spandex than Red 60, as evident from the darker appearance of the spandex samples. In fact, with Red 167, the spandex component exhibited such intense staining that the distinct coloration of the dyeing was obscured. As a result, the measured color strength values were slightly reduced, particularly at higher dye concentrations and lower dyeing temperatures (<130 °C) (Figure 10 and Figure 12) [25].

### 2.6. Color Fastness of Polyester/Spandex Blend Fabrics

Color fastness to washing of PET/spandex and PCP/spandex blends was evaluated at different dyeing temperatures. The assessments were conducted at medium depth levels (*f_k_* = 150 for Red 60 and 200 for Red 167) on the polyester fabrics (PET or PCP), obtained by adjusting the dye concentrations accordingly (Tables S13 and S14). For PET/spandex blends, dyeing at 90, 100, and 110 °C did not produce sufficient color strength on PET to reach the target depths. Therefore, only the results at 120 and 130 °C—where the required color strength was achieved—are presented and compared with those of the PCP/spandex blends.

The wash fastness results for PET/spandex and PCP/spandex blends dyed with Red 60 and Red 167 are summarized as grey scale ratings (1 = poor, 5 = excellent) in Table 2 and Table 3. In both cases, staining was observed on adjacent multifabrics, with nylon showing the most severe staining (Tables S15 and S16) [14]. PET/spandex blends, in particular, exhibited significant staining on nylon multifiber strips, especially when dyed with Red 167, where ratings of only 1–2 were recorded. This behavior is attributed to the substantial uptake of Red 167 by the spandex component in PET/spandex blends, leading to pronounced dye bleeding during washing. In contrast, PCP/spandex blends demonstrated relatively improved wash fastness, particularly with Red 167, where staining on adjacent nylon fabrics was reduced (ratings improving from 1–2 to 2–3) at lower dyeing temperatures of 110 and 120 °C. This improvement is primarily attributed to the lower staining tendency of the spandex component when blended with PCP fibers, which limits dye migration during laundering.

Color fastness to perspiration (both alkaline and acidic) was generally good for both PET/spandex and PCP/spandex blends, with ratings ranging from 3 to 3–4 [14]. However, PCP/spandex blends consistently achieved higher fastness ratings—typically by 0.5 to 1 grade—compared with PET/spandex blends under both perspiration conditions.

**Figure 13:**
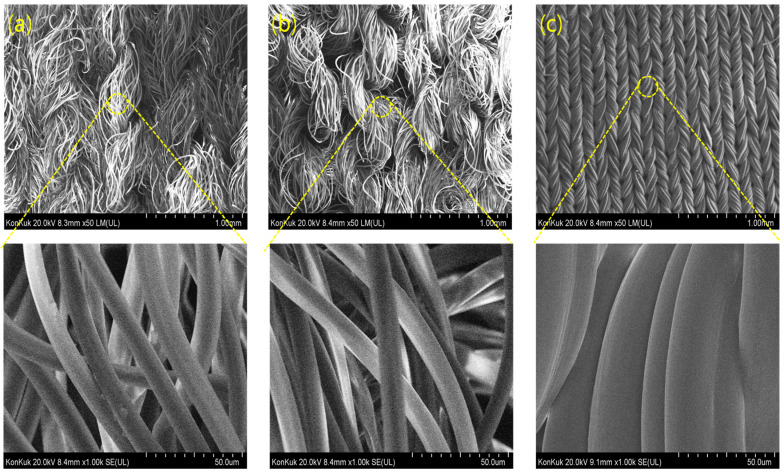
Microstructure of fabric samples: (**a**) PET, (**b**) PCP, and (**c**) spandex.

**Figure 14:**
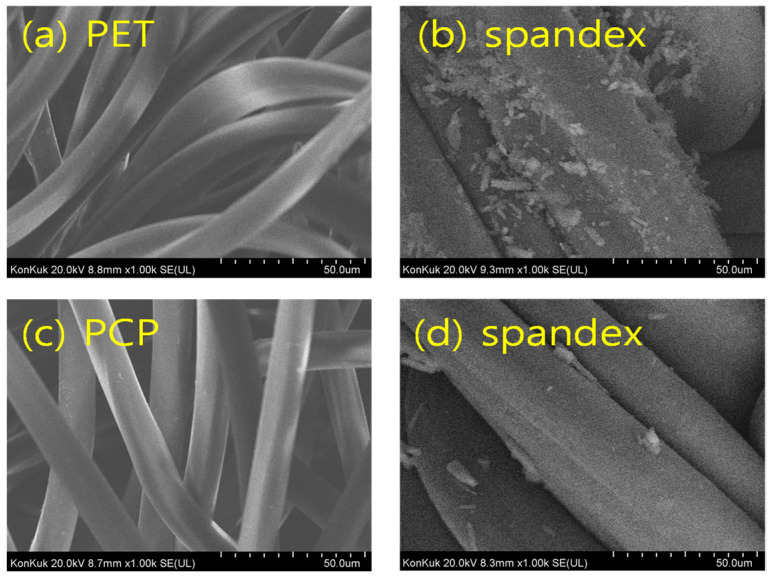
SEM images of polyester/spandex blends dyed with Red 60 at medium depths (*f_k_* = 150 on the polyesters component) in a same dyebath: (**a**,**b**) PET/spandex blend dyed at 120 °C and (**c**,**d**) PCP/spandex blend dyed at 110 °C.

**Figure 15:**
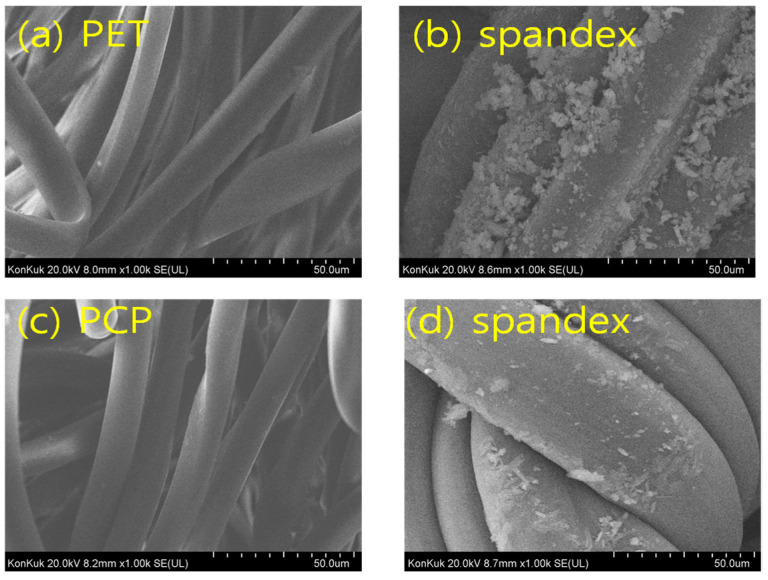
SEM images of polyester/spandex blends dyed with Red 167 at medium depths (*f_k_* = 200 on the polyesters component) in a same dyebath: (**a**,**b**) PET/spandex blend dyed at 120 °C and (**c**,**d**) PCP/spandex blend dyed at 110 °C.

The fastness results confirmed that PET/spandex blends exhibited heavier staining and lower ratings compared with PCP/spandex blends (Table 2 and Table 3). SEM analysis (Figure 13, Figure 14 and Figure 15) further revealed that disperse dyes were well absorbed within the polyester fibers but remained as non-absorbed surface deposits on spandex [4,22], a phenomenon more pronounced in PET/spandex samples. This effect can be attributed to the lower dye absorptivity of PET, which left excess dye available for deposition on spandex. By contrast, the copolymer structure of PCP facilitated higher dye uptake by the polyester component, thereby reducing spandex staining. Moreover, Red 167 produced more severe staining than Red 60, owing to its larger molecular size and limited absorption by polyester. Collectively, these results highlight that effective dye absorption by the polyester component is critical to minimizing spandex staining and enhancing overall fastness in polyester/spandex blends.

## 3. Materials and Methods

### 3.1. Materials

Regular polyester (PET; 75d/72f; density = 1.3865 g/cm^3^), poly(ethylene terephthalate-co-polyethylene glycol) (PCP; 75d/72f; density = 1.3715 g/cm^3^), and spandex (70d/5f; density = 0.9600 g/cm^3^) yarns were supplied by HUVIS Co., Ltd. (Daejeon, Republic of Korea). These yarns were knitted into single-jersey fabrics using a laboratory knitting machine (Kin-write Machine, Republic of Korea). Two disperse dyes, C.I. Disperse Red 60 (Red 60) and C.I. Disperse Red 167 (Red 167), were selected for this study. Their chemical structures and molecular weights are listed in Table 4. The dyes, along with a scouring agent (AZ-100) and a soaping agent, were obtained from a local textile dyeing company in South Korea. Additional chemicals—including acetic acid, *N,N*-dimethylformamide (DMF), sodium hydroxide (NaOH), and sodium dithionite (Na_2_S_2_O_4_)—were purchased from Daejung Chemicals and Metals Co., Ltd. (Gyeonggi-do, Republic of Korea). The dispersing agent (Dispertex DEG) was supplied by Donglim Chemicals (Incheon, Republic of Korea). Prior to dyeing, the PET, PCP, and spandex knitted fabrics were scoured in a solution containing 0.2 g/L NaOH and 1 g/L AZ-100 at 80 °C for 20 min. All dyes and chemicals were used without further purification.

### 3.2. Dyeing

**Figure 16:**
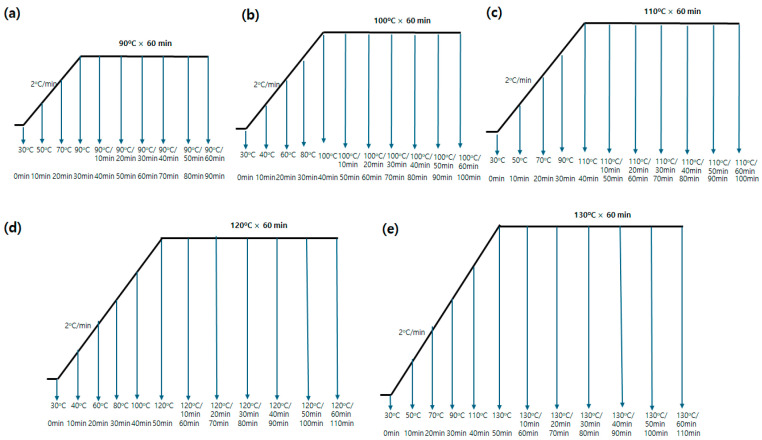
Dyeing profiles of polyester/spandex blends at different temperatures, with samples collected at 10 min intervals: (**a**) 90 °C, (**b**) 100 °C, (**c**) 110 °C, (**d**) 120 °C, and (**e**) 130 °C.

Dyeing was carried out using a laboratory infrared (IR) dyeing machine (DLS-6000, Daelim Starlet Co., Ltd., Republic of Korea). Each disperse dye (Red 60 or Red 167) was first dissolved in a minimal amount of acetone to prepare a 1% owf dye solution, which was then added to an aqueous dyebath containing Dispertex DEG (1.5 g/L). The bath pH was adjusted to 4.5–5.5 using acetic acid. Polyester (PET or PCP) and spandex fabrics were dyed together in the same dyebath at an 80:20 fabric weight ratio and a liquor ratio of 20:1. The dyeing cycle began at 30 °C, with the temperature increased at a rate of 2 °C/min until the target dyeing temperatures of 90, 100, 110, 120, or 130 °C were reached. The target temperature was then maintained for 60 min. During each dyeing run, fabric samples were collected every 10 min by temporarily halting the machine (Figure 16). After dyeing, all samples were thoroughly rinsed with distilled water and air-dried at room temperature.

The build-up properties of the polyester components (PET or PCP) and the degree of staining on the spandex components in polyester/spandex blends were evaluated. The blends were dyed at varying dye concentrations (1, 2, 3, 4, and 6% owf) and at different dyeing temperatures (90–130 °C) for a fixed duration of 60 min.

All dyeings under each condition were independently repeated three times (*n* = 3); plotted values are mean ± SD, with error bars showing replicate variability.

### 3.3. Color Strength Evaluation of Polyester and Spandex Fabrics

The color strength (*fₖ*) of polyester (PET or PCP) and spandex fabrics was evaluated using the Kubelka–Munk function, as expressed in Equation (2) [40]. Kubelka–Munk K/S values were measured over the wavelength range of 400–700 nm using a reflection spectrophotometer (X-Rite, Inc., Grand Rapids, MI, USA) with computer interfacing. Measurements were carried out under standardized colorimetric conditions: a 10° standard observer, illuminant D65, an aperture size of 10 mm, and with the specular component included. To ensure accuracy and reproducibility, each sample was measured three times, and the average value was calculated and reported.

(2) $$f_{k}\;=\;\sum_{\lambda =400}^{700}(K/S{)}_{\lambda }({\bar{x}}_{10,\lambda }+{\bar{y}}_{10,\lambda }+{\bar{z}}_{10,\lambda })$$

where ${\bar{x}}_{10,\lambda }$, ${\bar{y}}_{10,\lambda }$, and ${\bar{z}}_{10,\lambda }$ indicate the color matching functions based on a 10° visual field at each wavelength (ISO 7724/1–1984) [41]. 

### 3.4. Dyebath Exhaustion (%) Analysis of Disperse Dyes on Polyesters (PET or PCP) and Spandex Fabrics

Disperse dyes were separately extracted from each fabric component (PET, PCP, and spandex) using DMF at 90 °C until the samples were completely decolorized. The absorbance of the extracted dye solutions was then measured at the respective maximum absorption wavelengths using a UV–Vis spectrophotometer (Agilent 8000, Santa Clara, CA, USA). The amount of extracted dye was quantified using standard calibration curves prepared for each dye. The dyebath exhaustion of PET or PCP, as well as the degree of spandex staining, was calculated according to Equation (3):

(3) $$Exhaustion\left(\%\right)=\frac{D_{f}}{D_{b}}\times 100$$

where $D_{f}$ is the amount of dye extracted from the PET, PCP, or spandex fabric (mg), and $D_{b}$ is the initial amount of dye used in the dyebath (mg).

### 3.5. Diffusion Coefficient Measurement of Disperse Dyes on Polyesters (PET or PCP) and Spandex Fabrics

For determination of the diffusion coefficient, dyeing was performed using an infinite dyebath system, in which a small quantity of fiber was dyed in a large volume of dye solution. The initial dye concentration in the dyebath was fixed at 100 mg/g fabric for both Red 60 and Red 167. A dry fabric sample (0.05 g) was dyed in 200 mL of dye solution (material-to-liquor ratio = 1:4000) at constant temperatures of 100, 110, 120, and 130 °C. No dispersing agent was added to the dyebath; only acetic acid was included to maintain an acidic condition (pH 4.5–5.5). To ensure isothermal dyeing conditions, the fabric was first introduced into the dyebath at 90 °C. The bath was then rapidly heated to the target temperatures (100–130 °C) and maintained until equilibrium was reached. Dyebath samples were collected at predetermined time intervals during the isothermal process. After dyeing, the fabrics were rinsed thoroughly with water and dried at room temperature. Dye uptake was quantified by DMF extraction, as described previously.

The experimental dyeing rate curves (dye uptake versus time) were fitted to pseudo-first-order and pseudo-second-order kinetic models [42,43]. The pseudo-first-order kinetics model is expressed as:

(4) $$\frac{{dC}_{t}}{dt}=k_{1}(C_{e}-C_{t})$$

where k_1_ = rate constant (${min}^{-1}$), and $C_{t}$ and $C_{e}$ are dye uptake at time t (min) and at equilibrium, respectively (mg/g fabric).

The half dyeing time (t_1/2_), which represents the time required for the fiber to adsorb half of its equilibrium dye uptake, can be calculated by substituting $C_{t}$ = $C_{e}$/2 into Equation (4):

(5) $$t_{1/2}=\frac{ln2}{k_{1}}$$

where t_1/2_ = half dyeing time (min).

The pseudo-second-order kinetics is expressed as follows:

(6) $$\frac{{dC}_{t}}{dt}=k_{2}{\left(C_{e}-C_{t}\right)}^{2}$$

where $k_{2}$ = rate constant (mg/g min).

The t_1/2_ can be calculated by substituting $C_{t}$ = $C_{e}$/2 into Equation (6).

(7) $$t_{1/2}=\frac{1}{k_{2}C_{e}}$$

The diffusion coefficient, which is an important kinetics parameter, was calculated as follows using Hill’s Equation when $C_{t}$ = $C_{e}$/2 and t = t_1/2_ [42,43]:

(8) $$D=\frac{0.06292r^{2}}{t_{1/2}}$$

where D = diffusion coefficient (cm^2^/min), and r = fiber radius (cm).

### 3.6. Measurement of Distribution Ratio Between Polyester (PET or PCP) and Spandex Fabrics

The distribution of disperse dyes between the two components of the blends (polyester and spandex) was evaluated by calculating the distribution ratio before and after the reduction clearing (R/C) process, using Equation (9). For this purpose, blend fabrics were dyed with 1% owf dye at various temperatures for 60 min. Following dyeing, R/C was carried out at 80 °C for 20 min in a solution containing 2 g/L NaOH, 2 g/L Na_2_S_2_O_4_, and 1 g/L soaping agent.

(9) $$\mathrm{Distribution}\;\mathrm{ratio}=\frac{{CS}_{s}}{{CS}_{p}}=\frac{{DA}_{s}\times 100}{{DA}_{p}}$$

where ${CS}_{s}$ and ${CS}_{p}$ are the color strength of the spandex and the polyester (PET or PCP), and ${DA}_{s}$ and ${DA}_{p}$ are the dye amount (mg) extracted from the spandex and polyester components (PET or PCP), respectively.

The factor of 4 accounts for the weight ratio between polyester and spandex (80:20). Accordingly, the calculated distribution ratio represents the relative partitioning of dye between spandex and PET or PCP under identical dyebath exposure conditions.

### 3.7. Color Fastness Test

Prior to the fastness tests, the dyed blends underwent R/C and were subsequently heat-set at 180 °C for 30 s using a mini-tenter (DL-2015, Daelim Starlet Co., Ltd., Ansan, Republic of Korea). Color fastness to washing and perspiration was evaluated in accordance with ISO 105:C06 (A2S) [44] and ISO 105:E04 [45] standards, respectively. The degree of staining on multifiber adjacent fabrics was assessed using a spectrophotometer, following ISO 105:A04 [46] and ISO 105:A05 [47] protocols.

### 3.8. Observation of Adsorbed State on the Surface of Fibers

The sorbed state of dyes on the surfaces of polyester and spandex fibers was examined using a field-emission scanning electron microscope (FE-SEM, Hitachi SU8010, Tokyo, Japan).

## 4. Conclusions

In this study, the dyeing properties of chemically modified poly(ethylene terephthalate-co-polyethylene glycol) (PCP) blended with spandex were systematically evaluated and compared with those of conventional PET/spandex blends. Two disperse dyes of different molecular weights—C.I. Disperse Red 60 (low molecular weight) and C.I. Disperse Red 167 (high molecular weight)—were employed at dyeing temperatures of 90, 100, 110, 120, and 130 °C. Key dyeing parameters, including color strength, dyebath exhaustion, and distribution ratio, were examined.

The results demonstrated a strong correlation between increased dye uptake on the polyester components (PET or PCP) and reduced staining of the spandex component. PCP fibers, due to their chemical modification, exhibited superior dyeability—achieving higher color strength and dyebath exhaustion (≈80%)—compared with PET. As a result, spandex staining in PCP/spandex blends was significantly reduced, even at sub-conventional temperatures (<130 °C). In contrast, PET/spandex blends required higher dyeing temperatures (130 °C) to achieve only moderate dye uptake (50–60%) and to reduce spandex staining. Distribution ratio analysis further confirmed that spandex in PET/spandex blends was more prone to staining than in PCP/spandex blends. The reduction clearing (R/C) process effectively lowered distribution ratios, indicating successful removal of surface dyes from spandex. Between the two dyes, C.I. Disperse Red 167 exhibited a stronger tendency to stain spandex than C.I. Disperse Red 60, owing to its larger molecular size, which limited diffusion into the polyester matrix and increased its availability for spandex staining.

By balancing the dual objectives of maximizing dye uptake on polyester and minimizing spandex staining, a dyeing condition of 110 °C for 60 min was identified as optimal for PCP/spandex blends with both dyes. Under these conditions, PCP/spandex fabrics also exhibited higher color fastness compared with PET/spandex blends. Notably, PCP/spandex blends dyed with C.I. Disperse Red 167 showed an improvement of approximately one grade in wash fastness ratings at 110–120 °C, attributable to the reduced staining tendency of the spandex component, which minimized dye migration during washing.

Overall, these findings highlight PCP/spandex blends as a promising and energy-efficient alternative to conventional PET/spandex systems, enabling effective dyeing at lower temperatures while improving color fastness and offering potential energy savings in textile processing.

## Figures and Tables

**Table 1:** Diffusion coefficients of Red 60 and Red 167 on PET, PCP, and spandex fibers at different temperatures.

Samples	Temperature (°C)	Half Dyeing Time, t_1/2_ (min)		Diffusion Coefficient, D × 10^−9^ (cm^2^/min)	
		Red 60	Red 167	Red 60	Red 167
PET	100	102.42	33.01	0.16	0.51
	110	100.89	37.14	0.17	0.45
	120	26.24	25.90	0.64	0.65
	130	1.69	6.87	9.85	2.40
PCP	100	37.39	47.68	0.45	0.37
	110	16.01	3.67	1.05	3.67
	120	2.03	0.83	8.31	20.20
	130	0.31	N/A	54.85	N/A
Spandex	100	192.54	113.73	1.35	2.28
	110	135.91	25.21	1.91	10.29
	120	38.29	10.85	6.78	23.92
	130	33.16	6.85	7.83	37.90

**Table 2:** Wash fastness of polyester/spandex blends dyed with Red 60 at different temperatures.

Blends	Temp.	Change		Staining					
		Polyester	Spandex	W	A	P	N	C	A
PCP/spandex	90 °C	5	2	4–5	3–4	3	2–3	3–4	3–4
	100 °C	4–5	3	4	3–4	3	2–3	3–4	3–4
	110 °C	5	3–4	4	3–4	3	2–3	3–4	3–4
	120 °C	5	3	4–5	3–4	3	2–3	3–4	3–4
	130 °C	4–5	4–5	4–5	3–4	3	2–3	3–4	3–4
PET/spandex	120 °C	4	3–4	3–4	3–4	3	2	3	3
	130 °C	4–5	2	4	3–4	3	2	3	3

**Table 3:** Wash fastness of polyester/spandex blends dyed with Red 167 at different temperatures.

Blends	Temp.	Change		Staining					
		Polyester	Spandex	W	A	P	N	C	A
PCP/spandex	90 °C	5	2	3	3	2–3	1–2	2–3	3
	100 °C	4–5	2	3–4	3–4	2–3	2	3–4	3–4
	110 °C	4	3–4	4	4	3	2–3	3	3–4
	120 °C	4–5	2	4	4	3	2–3	3	3–4
	130 °C	4–5	2	4	4	3	2	3	3–4
PET/spandex	120 °C	3–4	1–2	2–3	2–3	2	1–2	2–3	2–3
	130 °C	5	2	3	3	2	1–2	2–3	2–3

**Table 4:** Chemical structures and molecular weights of the disperse dyes used in this study.

Dye (Code)	Chemical Structure	Molecular Weight (g/mol)
C.I. Disperse Red 60 (Red 60)	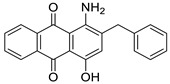	331.32
C.I. Disperse Red 67 (Red 167)	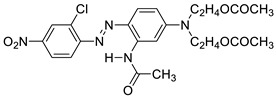	505.91

## Data Availability

All data for the manuscript are available upon request.

## Supplementary Materials

The following supporting information can be downloaded at: https://www.mdpi.com/article/10.3390/molecules30173578/s1, Table S1. Scanned images of PET/spandex (80:20) blends dyed with Red 60 at different temperatures in the same dyebath; Table S2. Scanned images of PET/spandex (80:20) blends dyed with Red 167 at different temperatures in same dyebath; Table S3. Scanned images of PCP/spandex (80:20) blends dyed with Red 60 at different temperatures in the same dyebath; Table S4. Scanned images of PCP/spandex (80:20) blends dyed with Red 167 at different temperatures in the same dyebath; Table S5. Logistic (regression) fit parameters for color strength of PET and staining on spandex in PET/spandex (80:20) blends dyed with Red 60 at different temperatures in the same dyebath; Table S6. Logistic (regression) fit parameters for color strength of PET and staining on spandex in PET/spandex (80:20) blends dyed with Red 167 at different temperatures in the same dyebath; Table S7. Logistic (regression) fit parameters for Color strength of PCP and staining on spandex in PCP/spandex (80:20) blends dyed with Red 60 at different temperatures in the same dyebath; Table S8. Logistic (regression) fit parameters for color strength of PCP and staining on spandex in PCP/spandex (80:20) blends dyed with Red 167 at different temperatures in the same dyebath; Table S9. Logistic (regression) fit parameters for dyebath exhaustion of PET and staining on spandex in PET/spandex (80:20) blends dyed with Red 60 at different temperatures in the same dyebath; Table S10. Logistic (regression) fit parameters for dyebath exhaustion of PET and staining on spandex in PET/spandex (80:20) blends dyed with Red 167 at different temperatures in the same dyebath; Table S11. Logistic (regression) fit parameters for dyebath exhaustion of PCP and staining on spandex in PCP/spandex (80:20) blends dyed with Red 60 at different temperatures in the same dyebath; Table S12. Logistic (regression) fit parameters for dyebath exhaustion of PCP and staining on spandex in PCP/spandex (80:20) blends dyed with Red 167 at different temperatures in the same dyebath; Table S13. Polyester/spandex dyed blends with medium depths (*f_k_* = 150) on the polyester fabrics (PET or PCP), achieved by applying appropriate concentrations of Red 60; Table S14. Polyester/spandex dyed blends with medium depths (*f_k_* = 200) on the polyester fabrics (PET or PCP), achieved by applying appropriate concentrations of Red 167; Table S15. Staining of multifabrics of polyester/spandex blends dyed with Red 60 at different temperatures; Table S16. Staining of multifabrics of polyester/spandex blends dyed with Red 167 at different temperatures; Figure S1. Logistic (regression) fit (using average values) for color strength of polyesters and staining on spandex in polyester/spandex (80:20) blends dyed with Red 60 at different temperatures in the same dyebath: (a–e) PET/spandex and (f–j) PCP/spandex; Figure S2. Logistic (regression) fit (using average values) for color strength of polyesters and staining on spandex in polyester/spandex (80:20) blends dyed with Red 167 at different temperatures in the same dyebath: (a–e) PET/spandex and (f–j) PCP/spandex; Figure S3. Logistic (regression) fit (using average values) for dyebath exhaustion of polyesters and staining on spandex in polyester/spandex (80:20) blends dyed with Red 60 at different temperatures in the same dyebath: (a–e) PET/spandex and (f–j) PCP/spandex; Figure S4. Logistic (regression) fit (using average values) for dyebath exhaustion of polyesters and staining on spandex in polyester/spandex (80:20) blends dyed with Red 167 at different temperatures in the same dyebath: (a–e) PET/spandex and (f–j) PCP/spandex.
