# Supplementary material for: Low-Temperature Dyeing of Chemically Modified PET/Spandex Blends: A Sustainable Approach for Enhanced Dyeability and Color Fastness
Source: Molecules. 2025 Sep 1;30(17):3578. doi: 10.3390/molecules30173578 (PMC12430684; doi:10.3390/molecules30173578)
Supplement: Supplementary file 1 [file molecules-30-03578-s001.zip › molecules-3801539-supplementary.pdf]

*Supplementary Materials*

# Low-Temperature Dyeing of Chemically Modified PET/Spandex Blends: A Sustainable Approach for Enhanced Dyeability and Color Fastness †

**Md Morshedur Rahman [1], Nazrul Hsan [2], Ingi Hong [1], Shekh Md Mamun Kabir [3], Seunga Choi [1], Youngdae Kim [1], Soohyun Kim [1] and Joonseok Koh [1,2,*]**

[1] Advanced Materials Program, Department of Materials Science and Engineering, Konkuk University, Seoul 05029, Republic of Korea; morshed133@gmail.com (M.M.R.); dlsrl3760@konkuk.ac.kr (I.H.); sachoi@konkuk.ac.kr (S.C.); ydkim0505@naver.com (Y.K.); modi7660@konkuk.ac.kr (S.K.)

[2] Department of Materials Science and Engineering, Konkuk University, Seoul 05029, Republic of Korea; nzsiddiqui90@gmail.com

[3] Department of Textile Materials Engineering, Bangladesh University of Textiles, Dhaka-1208, Bangladesh; head@tme.butex.edu.bd

* Correspondence: ccdjko@konkuk.ac.kr

† This article is an extended version of our earlier conference paper published in the *IOP Conference Series: Materials Science and Engineering* (Vol. 254, 082020, 2017; AUTEX 2017; doi:10.1088/1757-899X/254/8/082020).

**Table S1.** Scanned images of PET/spandex (80 :20) blends dyed with Red 60 at different temperatures in the same dyebath.

| | 30°C | 50°C | 70°C | 90°C | 90°C ×10' | 90°C ×20' | 90°C ×30' | 90°C ×40' | 90°C ×50' | 90°C ×60' | | |
|---|---|---|---|---|---|---|---|---|---|---|---|---|
| PET | | | | | | | | | | | | |
| Spandex | | | | | | | | | | | | |

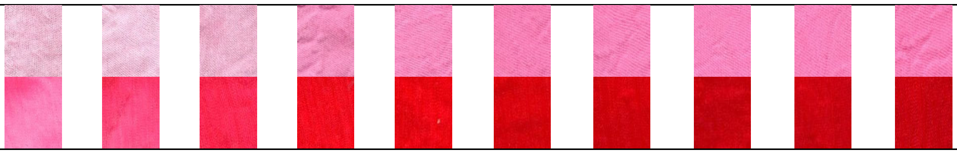

| | 30°C | 40°C | 60°C | 80°C | 100°C | 100°C ×10' | 100°C × 20' | 100°C × 30' | 100°C × 40' | 100°C × 50' | 100°C × 60' | |
|---|---|---|---|---|---|---|---|---|---|---|---|---|
| PET | | | | | | | | | | | | |
| Spandex | | | | | | | | | | | | |

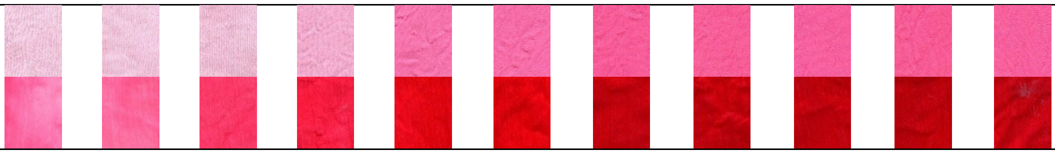

110°C

| | 30°C | 50°C | 70°C | 90°C | 110°C | 110°C ×10' | 110°C × 20' | 110°C × 30' | 110°C × 40' | 110°C × 50' | 110°C × 60' | |
|---|---|---|---|---|---|---|---|---|---|---|---|---|
| PET | | | | | | | | | | | | |
| Spandex | | | | | | | | | | | | |

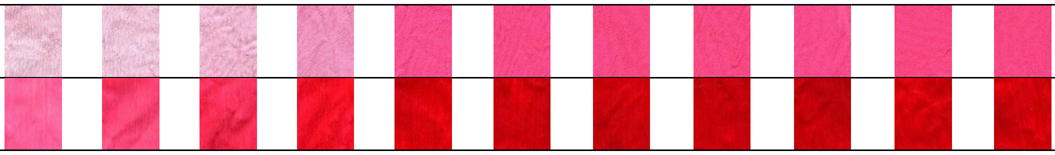

| | 30°C | 40°C | 60°C | 80°C | 100°C | 120°C | 120°C ×10' | 120°C × 20' | 120°C × 30' | 120°C × 40' | 120°C × 50' | 120°C × 60' |
|---|---|---|---|---|---|---|---|---|---|---|---|---|
| PET | | | | | | | | | | | | |
| Spandex | | | | | | | | | | | | |

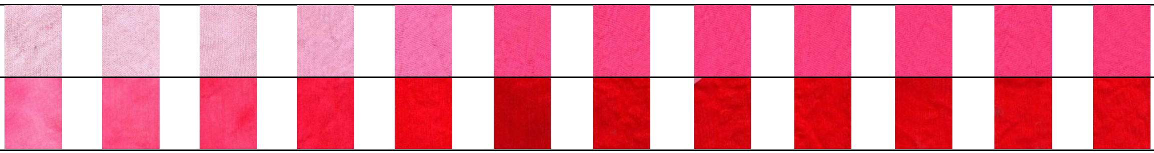

| | 30°C | 50°C | 70°C | 90°C | 110°C | 130°C | 130°C ×10' | 130°C × 20' | 130°C × 30' | 130°C × 40' | 130°C × 50' | 130°C × 60' |
|---|---|---|---|---|---|---|---|---|---|---|---|---|
| PET | | | | | | | | | | | | |
| Spandex | | | | | | | | | | | | |

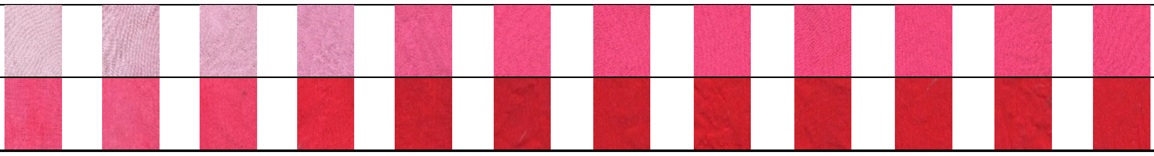

**Table S2.** Scanned images of PET/spandex (80 :20) blends dyed with Red 167 at different temperatures in same dyebath.

| | | | | | | | | | | | | |
|---|---|---|---|---|---|---|---|---|---|---|---|---|
| | 30°C | 50°C | 70°C | 90°C | 90°C ×10' | 90°C ×20' | 90°C ×30' | 90°C ×40' | 90°C ×50' | 90°C ×60' | | |
| PET | 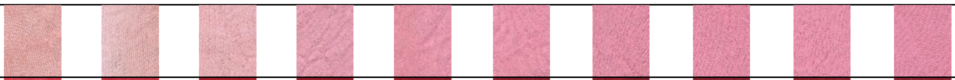 | | | | | | | | | | | |
| Spandex | 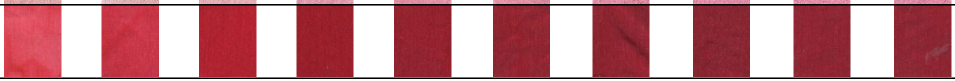 | | | | | | | | | | | |
| | 30°C | 40°C | 60°C | 80°C | 100°C | 100°C ×10' | 100°C × 20' | 100°C × 30' | 100°C × 40' | 100°C × 50' | 100°C × 60' | |
| PET | 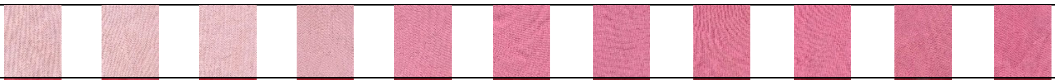 | | | | | | | | | | | |
| Spandex | 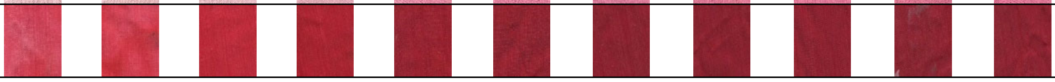 | | | | | | | | | | | |
| | 30°C | 50°C | 70°C | 90°C | 110°C | 110°C ×10' | 110°C × 20' | 110°C × 30' | 110°C × 40' | 110°C × 50' | 110°C × 60' | |
| PET | 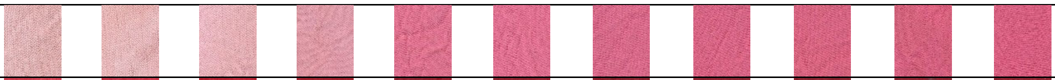 | | | | | | | | | | | |
| Spandex | 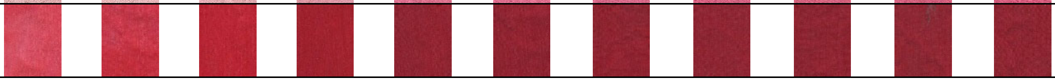 | | | | | | | | | | | |
| | 30°C | 40°C | 60°C | 80°C | 100°C | 120°C | 120°C ×10' | 120°C × 20' | 120°C × 30' | 120°C × 40' | 120°C × 50' | 120°C × 60' |
| PET | 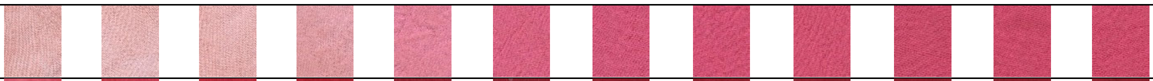 | | | | | | | | | | | |
| Spandex | 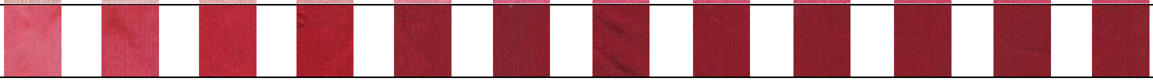 | | | | | | | | | | | |
| | 30°C | 50°C | 70°C | 90°C | 110°C | 130°C | 130°C ×10' | 130°C × 20' | 130°C × 30' | 130°C × 40' | 130°C × 50' | 130°C × 60' |
| PET | 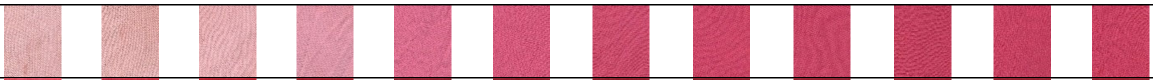 | | | | | | | | | | | |
| Spandex | 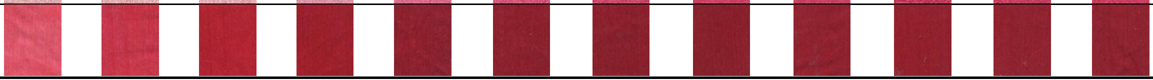 | | | | | | | | | | | |

**Table S3.** Scanned images of PCP/spandex (80 :20) blends dyed with Red 60 at different temperatures in the same dyebath.

| | | | | | | | | | | | |
|---|---|---|---|---|---|---|---|---|---|---|---|
| | 30°C | 50°C | 70°C | 90°C | 90°C ×10' | 90°C ×20' | 90°C ×30' | 90°C ×40' | 90°C ×50' | 90°C ×60' | |
| PCP | | | | | | | | | | | |
| Spandex | | | | | | | | | | | |
| | 30°C | 40°C | 60°C | 80°C | 100°C | 100°C ×10' | 100°C × 20' | 100°C × 30' | 100°C × 40' | 100°C × 50' | 100°C × 60' |
| PCP | | | | | | | | | | | |
| Spandex | | | | | | | | | | | |
| | 30°C | 50°C | 70°C | 90°C | 110°C | 110°C ×10' | 110°C × 20' | 110°C × 30' | 110°C × 40' | 110°C × 50' | 110°C × 60' |
| PCP | | | | | | | | | | | |
| Spandex | | | | | | | | | | | |
| | 30°C | 40°C | 60°C | 80°C | 100°C | 120°C | 120°C ×10' | 120°C × 20' | 120°C × 30' | 120°C × 40' | 120°C × 50' | 120°C × 60' |
| PCP | | | | | | | | | | | | |
| Spandex | | | | | | | | | | | | |
| | 30°C | 50°C | 70°C | 90°C | 110°C | 130°C | 130°C ×10' | 130°C × 20' | 130°C × 30' | 130°C × 40' | 130°C × 50' | 130°C × 60' |
| PCP | | | | | | | | | | | | |
| Spandex | | | | | | | | | | | | |

**Table S4.** Scanned images of PCP/spandex (80 :20) blends dyed with Red 167 at different temperatures in the same dyebath.

| | | | | | | | | | | | | |
|---|---|---|---|---|---|---|---|---|---|---|---|---|
| | 30°C | 50°C | 70°C | 90°C | 90°C ×10' | 90°C ×20' | 90°C ×30' | 90°C ×40' | 90°C ×50' | 90°C ×60' | | |
| PCP | | | | | | | | | | | | |
| Spandex | | | | | | | | | | | | |
| | 30°C | 40°C | 60°C | 80°C | 100°C | 100°C ×10' | 100°C × 20' | 100°C × 30' | 100°C × 40' | 100°C × 50' | 100°C × 60' | |
| PCP | | | | | | | | | | | | |
| Spandex | | | | | | | | | | | | |
| | 30°C | 50°C | 70°C | 90°C | 110°C | 110°C ×10' | 110°C × 20' | 110°C × 30' | 110°C × 40' | 110°C × 50' | 110°C × 60' | |
| PCP | | | | | | | | | | | | |
| Spandex | | | | | | | | | | | | |
| | 30°C | 40°C | 60°C | 80°C | 100°C | 120°C | 120°C ×10' | 120°C × 20' | 120°C × 30' | 120°C × 40' | 120°C × 50' | 120°C × 60' |
| PCP | | | | | | | | | | | | |
| Spandex | | | | | | | | | | | | |
| | 30°C | 50°C | 70°C | 90°C | 110°C | 130°C | 130°C ×10' | 130°C × 20' | 130°C × 30' | 130°C × 40' | 130°C × 50' | 130°C × 60' |
| PCP | | | | | | | | | | | | |
| Spandex | | | | | | | | | | | | |

**Table S5.** Logistic (regression) fit parameters for color strength of PET and staining on spandex in PET/spandex (80 :20) blends dyed with Red 60 at different temperatures in the same dyebath.

| Parameters | PET | | | | |
|---|---|---|---|---|---|
| | **90°C** | **100°C** | **110°C** | **120°C** | **130°C** |
| $A_1$ | 2.215±0.899 | 2.019±0.860 | 3.727±0.753 | 2.586±0.932 | 6.601±1.580 |
| $A_2$ | 8.521 ±0.565 | 16.918±1.013 | 25.305±0.538 | 34.311±0.877 | 37.718±1.424 |
| $x_0$ | 26.345±3.920 | 40.468±2.840 | 34.643±1.216 | 45.285±1.236 | 43.512±2.051 |
| $p$ | 7.287±5.646 | 4.773±1.492 | 15.443±3.646 | 7.527±1.340 | 7.427±2.294 |
| $R^2$ (COD) | 0.882 | 0.969 | 0.989 | 0.990 | 0.971 |
| Adj. $R^2$ | 0.823 | 0.956 | 0.984 | 0.986 | 0.961 |
| **Parameters** | **Spandex** | | | | |
| | **90°C** | **100°C** | **110°C** | **120°C** | **130°C** |
| $A_1$ | 13.122 ±6.268 | 18.093±6.548 | 23.826±10.705 | 22.450±11.955 | 46.232±10.299 |
| $A_2$ | 296.723±6.390 | 291.470±5.337 | 281.747±6.833 | 252.991±6.935 | 258.181±5.855 |
| $x_0$ | 27.591±0.886 | 33.528±0.860 | 26.961±1.256 | 28.714±1.324 | 28.474±1.324 |
| $p$ | 3.322±0.342 | 5.586±0.715 | 5.293±1.052 | 8.722±4.158 | 10.034±5.955 |
| $R^2$ (COD) | 0.996 | 0.995 | 0.987 | 0.977 | 0.980 |
| Adj. $R^2$ | 0.995 | 0.993 | 0.981 | 0.968 | 0.972 |

**Table S6.** Logistic (regression) fit parameters for color strength of PET and staining on spandex in PET/spandex (80 :20) blends dyed with Red 167 at different temperatures in the same dyebath.

| Parameters | PET | | | | |
|---|---|---|---|---|---|
| | **90°C** | **100°C** | **110°C** | **120°C** | **130°C** |
| $A_1$ | 5.145±0.183 | 2.078±0.383 | 3.487±1.467 | 3.549±0.495 | 3.726±1.192 |
| $A_2$ | 7.669±0.173 | 12.094±0.369 | 24.530±2.867 | 39.488±0.689 | 62.478±2.229 |
| $x_0$ | 39.293±2.244 | 38.831±1.585 | 42.590±5.500 | 51.373±0.821 | 54.163±1.575 |
| $p$ | 9.819±5.919 | 5.788±1.225 | 3.192 ± 1.234 | 5.520±0.452 | 4.598±0.570 |
| $R^2$ (COD) | 0.953 | 0.986 | 0.956 | 0.997 | 0.994 |
| Adj. $R^2$ | 0.930 | 0.980 | 0.937 | 0.996 | 0.992 |
| **Parameters** | **Spandex** | | | | |
| | **90°C** | **100°C** | **110°C** | **120°C** | **130°C** |
| $A_1$ | 41.604±20.390 | 33.042±9.711 | 58.531±7.651 | 49.433±14.256 | 56.044±21.705 |
| $A_2$ | 338.993±12.563 | 335.159±6.817 | 325.250±4.431 | 308.565±7.432 | 318.088±9.475 |
| $x_0$ | 17.583±1.874 | 24.043±1.043 | 20.623±0.663 | 21.700±1.218 | 17.409±2.051 |
| $p$ | 2.932±0.754 | 3.544±0.501 | 4.882±0.803 | 5.752±1.777 | 3.551±1.155 |
| $R^2$ (COD) | 0.976 | 0.993 | 0.994 | 0.975 | 0.959 |
| Adj. $R^2$ | 0.964 | 0.990 | 0.991 | 0.965 | 0.944 |

**Table S7.** Logistic (regression) fit parameters for Color strength of PCP and staining on spandex in PCP/spandex (80 :20) blends dyed with Red 60 at different temperatures in the same dyebath.

| Parameters | PCP | | | | |
|---|---|---|---|---|---|
| | **90°C** | **100°C** | **110°C** | **120°C** | **130°C** |
| $A_1$ | 4.570±0.883 | 2.444±0.996 | 4.220±2.209 | 4.166±1.976 | 6.450±2.143 |
| $A_2$ | 56.081±0.634 | 59.540±0.722 | 65.803±1.562 | 61.171±1.315 | 61.354±1.403 |
| $x_0$ | 29.231±0.412 | 33.805±0.516 | 30.032±5.282 | 33.427±1.004 | 30.395±1.776 |
| $p$ | 8.900±1.510 | 11.322±1.191 | 69.742±1.117 | 11.746±2.598 | 49.874±2.227 |
| $R^2$ (COD) | 0.997 | 0.997 | 0.987 | 0.988 | 0.984 |
| Adj. $R^2$ | 0.996 | 0.996 | 0.982 | 0.983 | 0.978 |
| **Parameters** | **Spandex** | | | | |
| | **90°C** | **100°C** | **110°C** | **120°C** | **130°C** |
| $A_1$ | 27.299±3.774 | 20.319±6.347 | 28.871±9.000 | 26.728±5.857 | 17.379±8.252 |
| $A_2$ | 241.148±2.714 | 209.920±4.382 | 202.507±5.074 | 225.898±4.093 | 242.911±6.260 |
| $x_0$ | 28.526±0.516 | 30.238±0.892 | 24.571±1.334 | 31.281±1.035 | 27.965±1.405 |
| $p$ | 6.227±0.639 | 7.496±1.804 | 7.069±1.708 | 4.942±0.721 | 3.405±0.533 |
| $R^2$ (COD) | 0.997 | 0.990 | 0.980 | 0.992 | 0.989 |
| Adj. $R^2$ | 0.996 | 0.986 | 0.972 | 0.989 | 0.986 |

**Table S8.** Logistic (regression) fit parameters for color strength of PCP and staining on spandex in PCP/spandex (80 :20) blends dyed with Red 167 at different temperatures in the same dyebath.

| Parameters | PCP | | | | |
|---|---|---|---|---|---|
| | **90°C** | **100°C** | **110°C** | **120°C** | **130°C** |
| $A_1$ | 3.112±1.424 | 0.665±5.471 | 4.055±2.090 | 3.142±2.458 | 4.102±1.047 |
| $A_2$ | 68.999±1.060 | 94.418±5.026 | 117.930±1.787 | 120.726±1.841 | 119.481±0.727 |
| $x_0$ | 28.629±0.663 | 37.170±2.341 | 36.262±0.687 | 38.092±0.720 | 33.843±0.290 |
| $p$ | 5.691±0.669 | 5.648±1.776 | 6.153±0.615 | 7.832±1.004 | 7.195±0.363 |
| $R^2$ (COD) | 0.996 | 0.969 | 0.996 | 0.995 | 0.999 |
| Adj. $R^2$ | 0.995 | 0.957 | 0.995 | 0.993 | 0.998 |
| **Parameters** | **Spandex** | | | | |
| | **90°C** | **100°C** | **110°C** | **120°C** | **130°C** |
| $A_1$ | 54.401±17.604 | 63.584±10.982 | 58.642±14.635 | 29.710±13.137 | 42.400±9.664 |
| $A_2$ | 250.421±6.965 | 340.907±21.180 | 282.789±7.918 | 313.344±9.855 | 306.996±4.102 |
| $x_0$ | 12.943±1.586 | 25.641±3.283 | 15.641±1.671 | 21.125±1.685 | 15.642±0.852 |
| $p$ | 5.038±1.923 | 1.609±0.299 | 2.691±0.604 | 2.354±0.404 | 3.228±0.401 |
| $R^2$ (COD) | 0.963 | 0.987 | 0.975 | 0.985 | 0.991 |
| Adj. $R^2$ | 0.945 | 0.982 | 0.964 | 0.980 | 0.988 |

**Table S9.** Logistic (regression) fit parameters for dyebath exhaustion of PET and staining on spandex in PET/spandex (80 :20) blends dyed with Red 60 at different temperatures in the same dyebath.

| **Parameters** | **PET** | | | | |
|---|---|---|---|---|---|
| | **90°C** | **100°C** | **110°C** | **120°C** | **130°C** |
| $A_1$ | 13.916±3.267 | 12.919±1.083 | 10.641±1.369 | 6.874±1.562 | 5.772±1.139 |
| $A_2$ | 26.625±1.914 | 30.222±1.026 | 50.884±0.983 | 52.864±1.293 | 58.613±0.987 |
| $x_0$ | 5.421±5.603 | 37.007±2.592 | 33.939±1.065 | 42.475±1.065 | 42.020±0.839 |
| $p$ | 2.915±0.505 | 5.288±1.757 | 13.475±2.951 | 14.641±4.421 | 7.318±0.965 |
| $R^2$ (COD) | 0.403 | 0.965 | 0.989 | 0.986 | 0.994 |
| Adj. $R^2$ | 0.360 | 0.951 | 0.985 | 0.981 | 0.993 |
| **Parameters** | **Spandex** | | | | |
| | **90°C** | **100°C** | **110°C** | **120°C** | **130°C** |
| $A_1$ | 5.638±1.399 | 4.563±0.913 | 5.174±1.293 | 5.500±1.756 | 4.564±1.572 |
| $A_2$ | 64.119±2.620 | 71.540±1.170 | 56.821±1.107 | 50.428±1.434 | 41.225±1.046 |
| $x_0$ | 44.497±1.539 | 46.067±0.691 | 35.693±0.941 | 40.272±4.230 | 32.316±1.133 |
| $p$ | 4.608±0.680 | 5.916±0.483 | 5.884±0.792 | 7.970±1.235 | 10.753±3.451 |
| $R^2$ (COD) | 0.994 | 0.997 | 0.994 | 0.981 | 0.981 |
| Adj. $R^2$ | 0.991 | 0.997 | 0.992 | 0.974 | 0.974 |

**Table S10.** Logistic (regression) fit parameters for dyebath exhaustion of PET and staining on spandex in PET/spandex (80 :20) blends dyed with Red 167 at different temperatures in the same dyebath.

| **Parameters** | **PET** | | | | |
|---|---|---|---|---|---|
| | **90°C** | **100°C** | **110°C** | **120°C** | **130°C** |
| $A_1$ | 6.004±2.425 | 7.974±2.185 | 7.601±1.329 | 11.022±1.123 | 7.652±3.487 |
| $A_2$ | 13.285±0.996 | 14.627±0.782 | 27.276±1.741 | 43.941±3.485 | 90.105±48.201 |
| $x_0$ | 8.359±8.230 | 16.054±29.833 | 34.761±3.678 | 54.406±5.018 | 85.737±65.525 |
| $p$ | 4.186±21.297 | 10.216±84.804 | 3.168±1.041 | 2.902±0.628 | 1.514±0.657 |
| $R^2$ (COD) | 0.580 | 0.676 | 0.965 | 0.985 | 0.964 |
| Adj. $R^2$ | 0.370 | 0.537 | 0.950 | 0.980 | 0.951 |
| **Parameters** | **Spandex** | | | | |
| | **90°C** | **100°C** | **110°C** | **120°C** | **130°C** |
| $A_1$ | 4.115±1.480 | 6.447±2.223 | 4.722±1.958 | 6.750±4.228 | 6.333±7.218 |
| $A_2$ | 96.087±1.714 | 91.851±1.935 | 78.786±1.115 | 69.714±3.452 | 60.375±4.420 |
| $x_0$ | 33.825±0.713 | 39.878±0.858 | 27.105±0.754 | 39.884±1.406 | 30.044±0.000 |
| $p$ | 4.173±0.348 | 8.895±1.6990 | 8.833±1.753 | 83.499±1.021 | 219.33±0.000 |
| $R^2$ (COD) | 0.998 | 0.99327 | 0.994 | 0.946 | 0.841 |
| Adj. $R^2$ | 0.997 | 0.99038 | 0.992 | 0.926 | 0.781 |

**Table S11.** Logistic (regression) fit parameters for dyebath exhaustion of PCP and staining on spandex in PCP/spandex (80 :20) blends dyed with Red 60 at different temperatures in the same dyebath.

| **Parameters** | **PCP** | | | | |
|---|---|---|---|---|---|
| | **90°C** | **100°C** | **110°C** | **120°C** | **130°C** |
| $A_1$ | 12.523±0.654 | 6.895±2.204 | 22.278±2.789 | 22.297±1.721 | 9.666±2.933 |
| $A_2$ | 69.839±0.516 | 76.465±1.581 | 84.285±1.966 | 82.586±1.138 | 75.625±1.794 |
| $x_0$ | 30.794±0.313 | 32.156±0.874 | 30.048±5.282 | 32.594±0.845 | 30.020±0.000 |
| $p$ | 7.456±0.600 | 12.739±3.829 | 84.567±9.121 | 13.605±3.467 | 149.683±0.000 |
| $R^2$ (COD) | 0.998 | 0.990 | 0.980 | 0.991 | 0.979 |
| Adj. $R^2$ | 0.998 | 0.986 | 0.972 | 0.988 | 0.971 |
| **Parameters** | **Spandex** | | | | |
| | **90°C** | **100°C** | **110°C** | **120°C** | **130°C** |
| $A_1$ | 4.303±0.443 | 2.749±0.5110 | 9.333±0.441 | 4.360±1.200 | 2.497±0.925 |
| $A_2$ | 34.267±0.555 | 23.952±0.409 | 19.285±0.289 | 28.021±1.270 | 35.714±1.572 |
| $x_0$ | 34.679±0.698 | 34.353±0.838 | 30.024±0.000 | 43.186±2.498 | 41.010±2.118 |
| $p$ | 4.016±0.311 | 6.334±0.835 | 153.285±0.000 | 5.133±1.404 | 2.8115±0.396 |
| $R^2$ (COD) | 0.998 | 0.994 | 0.981 | 0.973 | 0.992 |
| Adj. $R^2$ | 0.997 | 0.992 | 0.973 | 0.963 | 0.990 |

**Table S12.** Logistic (regression) fit parameters for dyebath exhaustion of PCP and staining on spandex in PCP/spandex (80 :20) blends dyed with Red 167 at different temperatures in the same dyebath.

| **Parameters** | **PCP** | | | | |
|---|---|---|---|---|---|
| | **90°C** | **100°C** | **110°C** | **120°C** | **130°C** |
| $A_1$ | 8.285±2.024 | 6.303±2.499 | 3.884±1.331 | 8.617±3.123 | 15.649±3.522 |
| $A_2$ | 63.028±1.260 | 69.012±1.837 | 78.456±1.022 | 74.095±1.640 | 71.842±2.412 |
| $x_0$ | 26.234±1.014 | 33.910±1.187 | 29.870±0.634 | 26.242±1.308 | 33.356±1.926 |
| $p$ | 7.543±1.528 | 9.598±2.210 | 4.555±0.393 | 7.970±2.151 | 7.544±2.703 |
| $R^2$ (COD) | 0.991 | 0.985 | 0.997 | 0.982 | 0.962 |
| Adj. $R^2$ | 0.986 | 0.979 | 0.996 | 0.975 | 0.948 |
| **Parameters** | **Spandex** | | | | |
| | **90°C** | **100°C** | **110°C** | **120°C** | **130°C** |
| $A_1$ | 4.517±1.584 | 5.000±1.895 | 3.556±3.640 | 4.500±3.824 | 5.333±2.772 |
| $A_2$ | 39.400±0.957 | 39.285±1.340 | 31.014±1.978 | 28.777±1.912 | 32.625±1.815 |
| $x_0$ | 23.752±1.120 | 30.382±2.716 | 23.118±3.386 | 20.192±1.396 | 29.862±2.321 |
| $p$ | 7.689±1.706 | 59.151±4.172 | 9.954±8.264 | 45.019±3.173 | 64.780±1.093 |
| $R^2$ (COD) | 0.986 | 0.972 | 0.885 | 0.819 | 0.898 |
| Adj. $R^2$ | 0.979 | 0.960 | 0.836 | 0.751 | 0.861 |

**Table S13.** Polyester/spandex dyed blends with medium depths ($f_k$ = 150) on the polyester fabrics (PET or PCP), achieved by applying appropriate concentrations of Red 60.

| Temperature | PET/spandex | | EDP/Spandex | |
|---|---|---|---|---|
| | PET | Spandex | EDP | Spandex |
| 90°C | N/A | N/A | 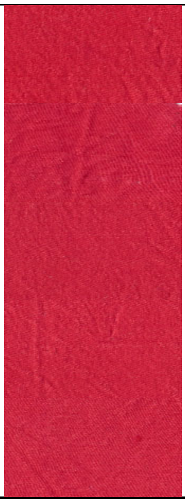 | 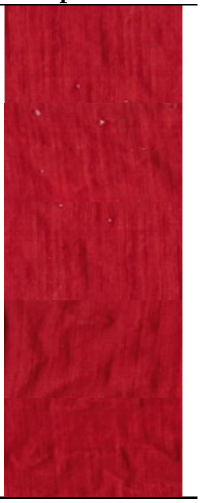 |
| 100°C | N/A | N/A | | |
| 110°C | N/A | N/A | | |
| 120°C | 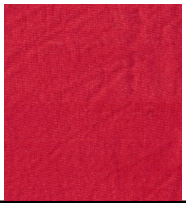 | 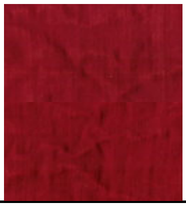 | | |
| 130°C | | | | |

**Table S14.** Polyester/spandex dyed blends with medium depths ($f_k$ = 200) on the polyester fabrics (PET or PCP), achieved by applying appropriate concentrations of Red 167.

| Temper-atures | PET/Spandex | | EDP/Spandex | |
|---|---|---|---|---|
| | PET | Spandex | EDP | Spandex |
| 90°C | N/A | N/A | 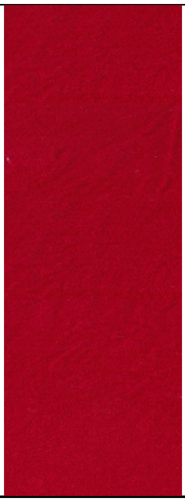 | 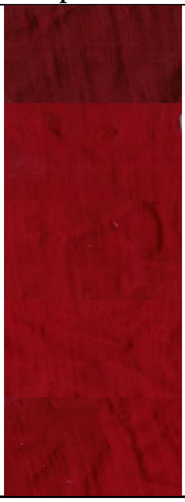 |
| 100°C | N/A | N/A | | |
| 110°C | N/A | N/A | | |
| 120°C | 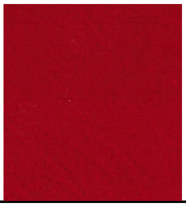 | 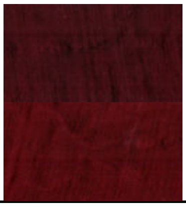 | | |
| 130°C | | | | |

**Table S15.** Staining of multifabrics of polyester/spandex blends dyed with Red 60 at different temperatures.

| Multifabrics | PET/Spandex | | PCP/spandex | | | | |
|---|---|---|---|---|---|---|---|
| | 120°C | 130°C | 90°C | 100°C | 110°C | 120°C | 130°C |
| Wool | | | | | | | |
| Acrylic | | | | | | | |
| PET | | | | | | | |
| Nylon | | | | | | | |
| Cotton | | | | | | | |
| Acetate | | | | | | | |

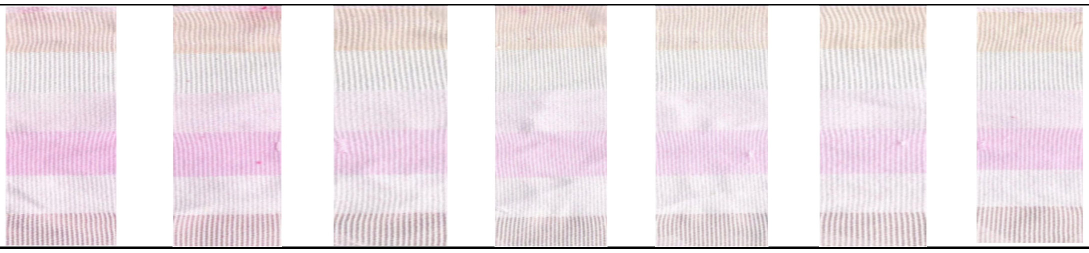

**Table S16.** Staining of multifabrics of polyester/spandex blends dyed with Red 167 at different temperatures.

| Multifabrics | PET/Spandex | | PCP/spandex | | | | |
|---|---|---|---|---|---|---|---|
| | 120°C | 130°C | 90°C | 100°C | 110°C | 120°C | 130°C |
| Wool | | | | | | | |
| Acrylic | | | | | | | |
| PET | | | | | | | |
| Nylon | | | | | | | |
| Cotton | | | | | | | |
| Acetate | | | | | | | |

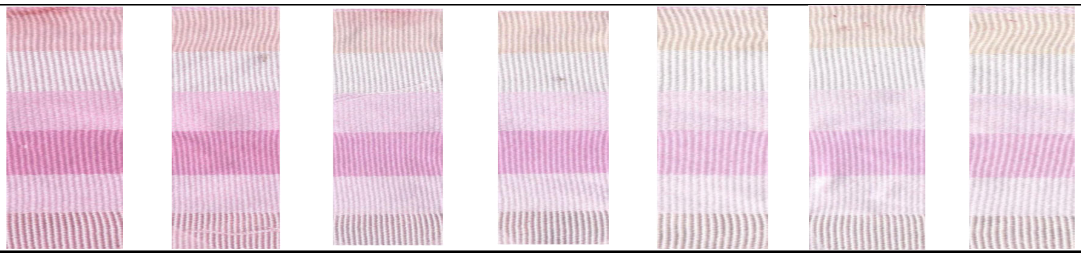

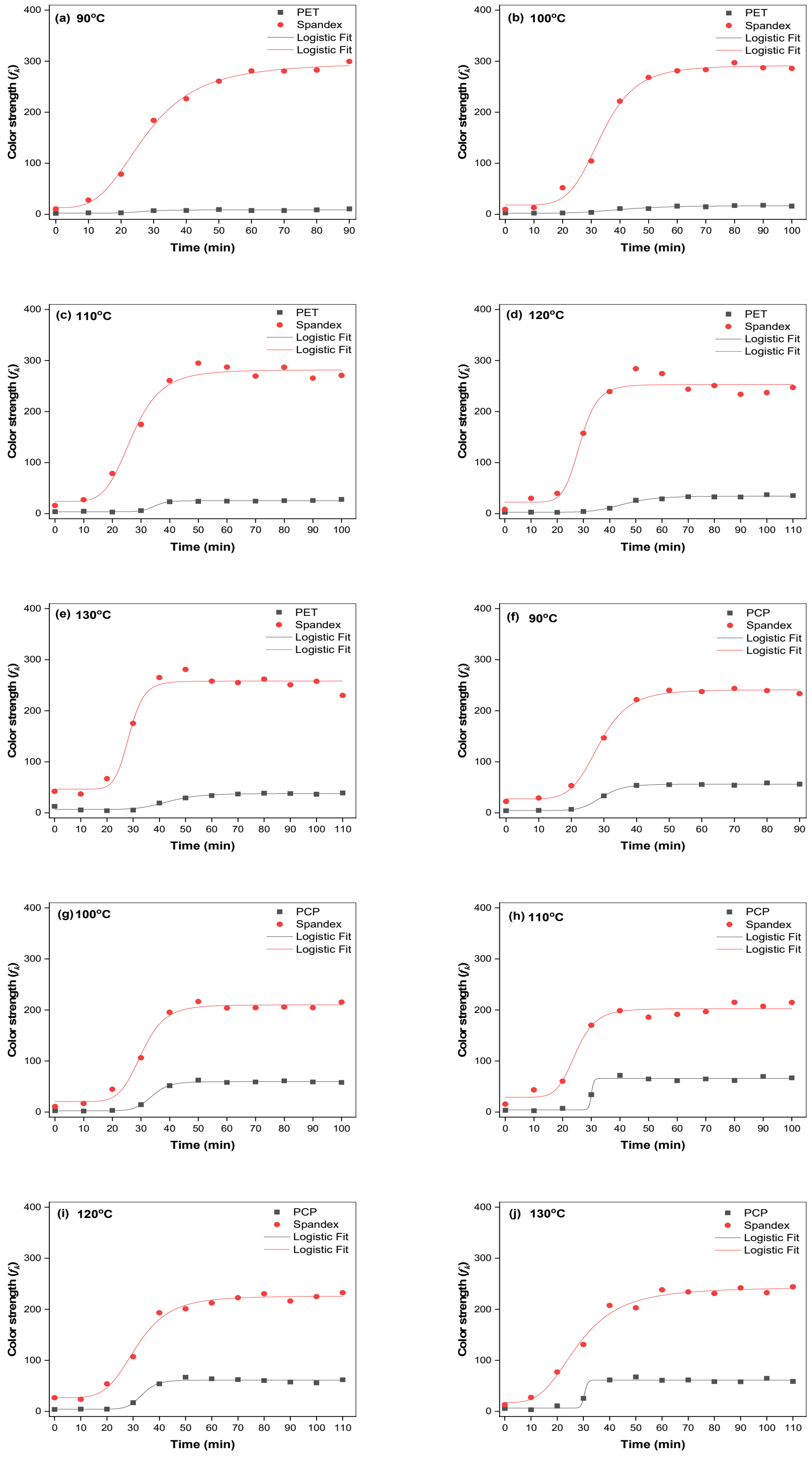


**Figure S1.** Logistic (regression) fit (using average values) for color strength of polyesters and staining on spandex in polyester/spandex (80:20) blends dyed with Red 60 at different temperatures in the same dyebath: (a-e) PET/spandex and (f-j) PCP/spandex.

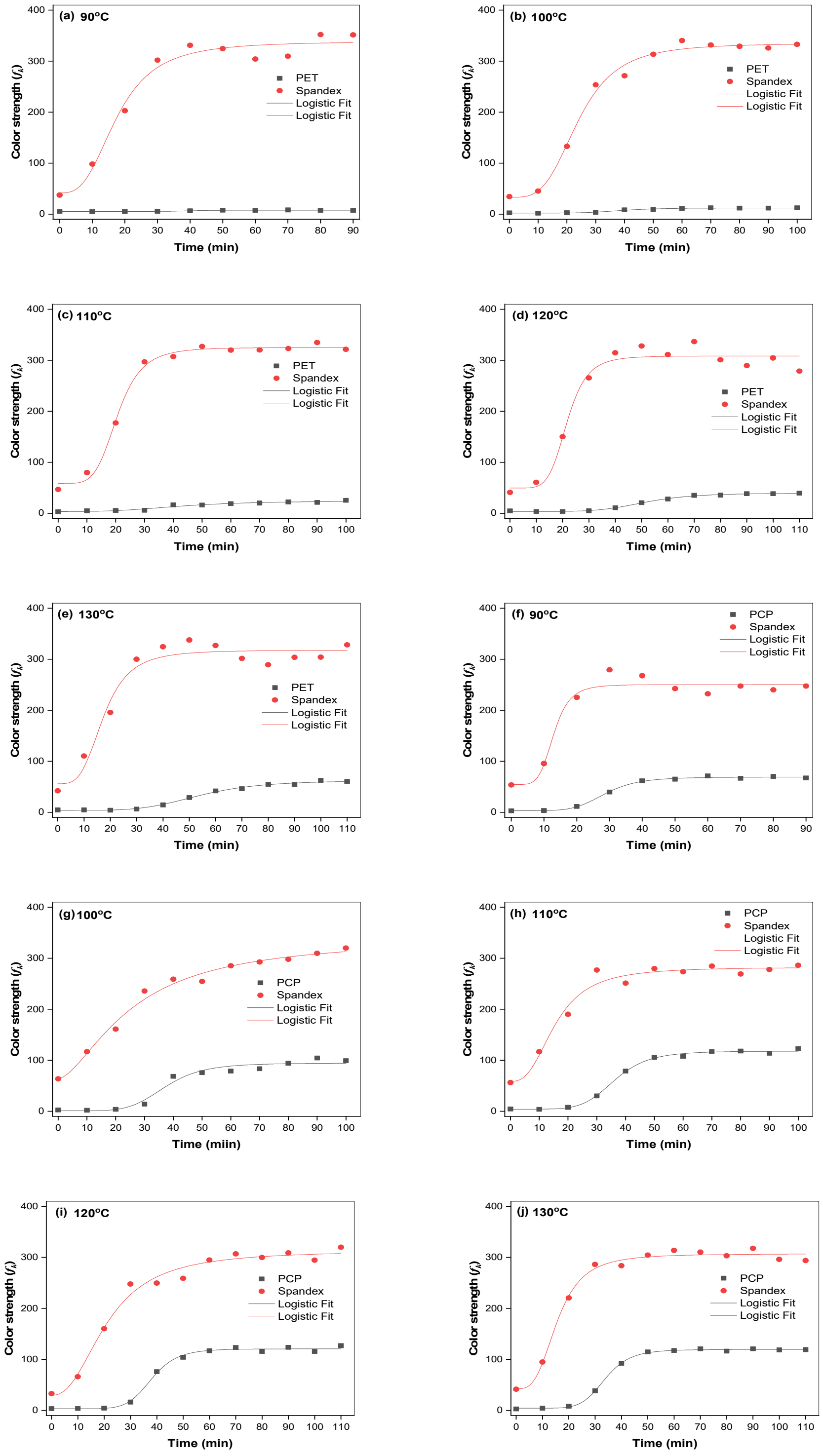


**Figure S2.** Logistic (regression) fit (using average values) for color strength of polyesters and staining on spandex in polyester/spandex (80:20) blends dyed with Red 167 at different temperatures in the same dyebath: (a-e) PET/spandex and (f-j) PCP/spandex.

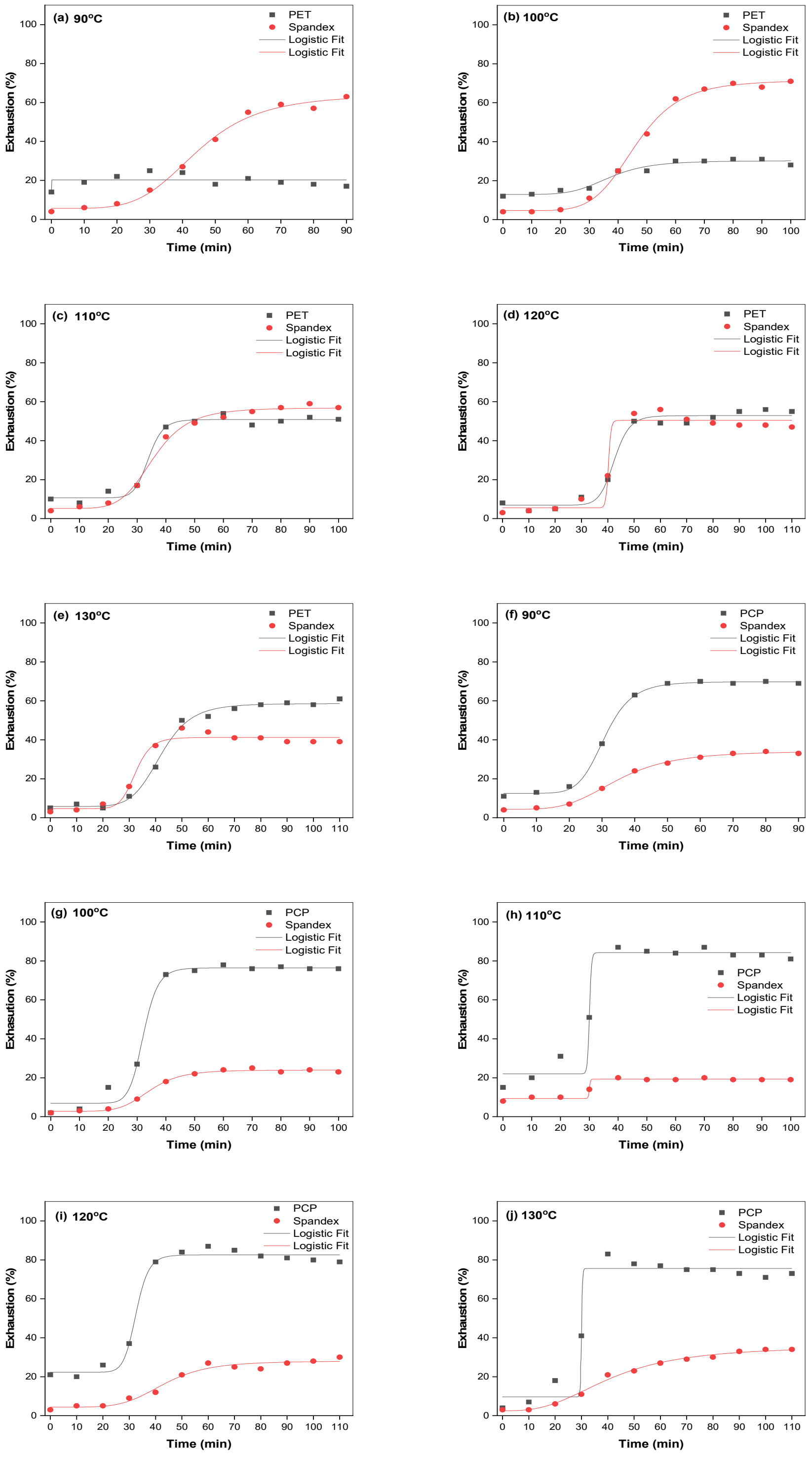


**Figure S3.** Logistic (regression) fit (using average values) for dyebath exhaustion of polyesters and staining on spandex in polyester/spandex (80:20) blends dyed with Red 60 at different temperatures in the same dyebath: (a-e) PET/spandex and (f-j) PCP/spandex.

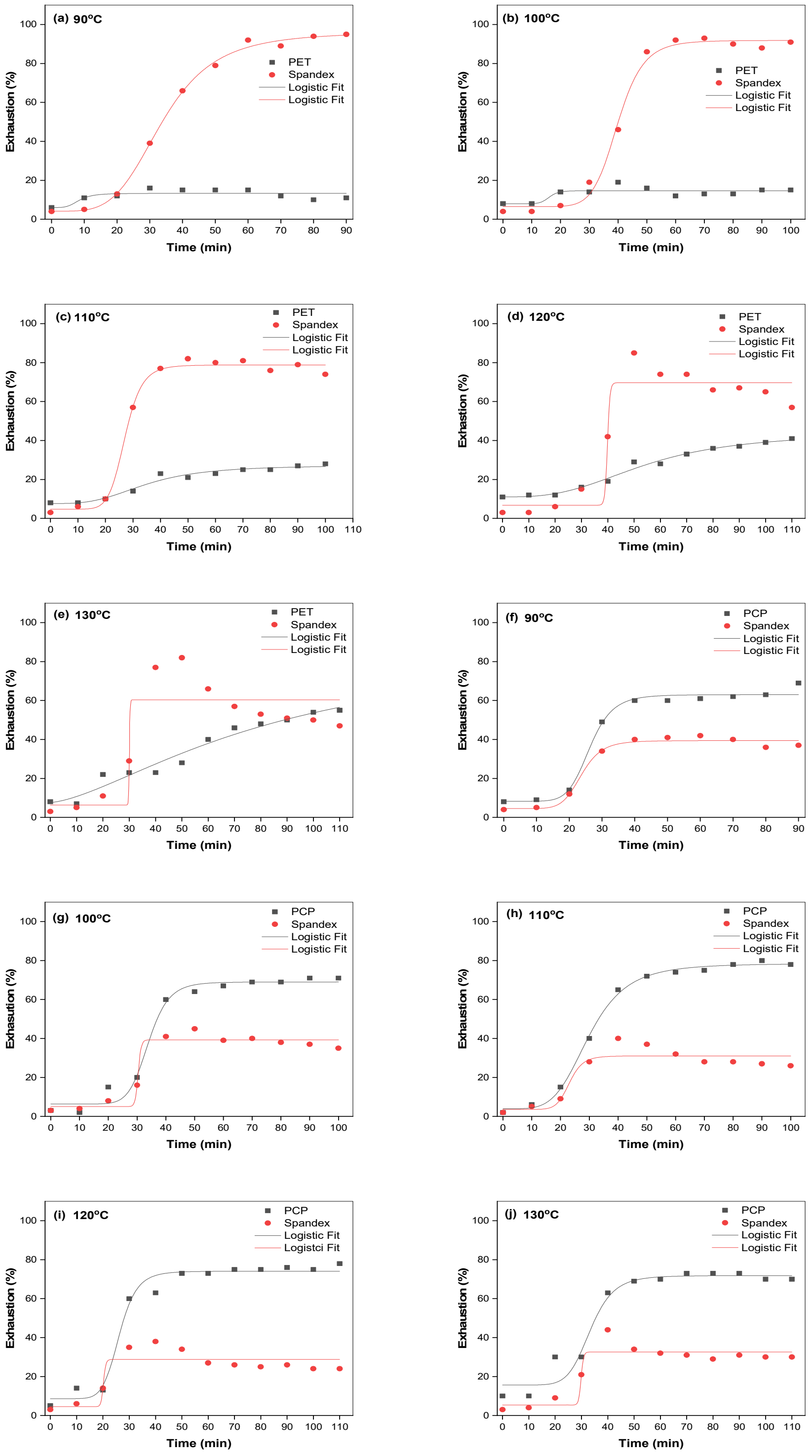


**Figure S4.** Logistic (regression) fit (using average values) for dyebath exhaustion of polyesters and staining on spandex in polyester/spandex (80:20) blends dyed with Red 167 at different temperatures in the same dyebath: (a-e) PET/spandex and (f-j) PCP/spandex.
